# Biomaterials combined with ADSCs for bone tissue engineering: current advances and applications

**DOI:** 10.1093/rb/rbad083

**Published:** 2023-09-12

**Authors:** Yiping Song, Ning Wang, Huixin Shi, Dan Zhang, Qiang Wang, Shu Guo, Shude Yang, Jia Ma

**Affiliations:** Department of Plastic Surgery, The First Hospital of China Medical University, Shenyang 110001, China; Department of Plastic Surgery, The First Hospital of China Medical University, Shenyang 110001, China; Department of Plastic Surgery, The First Hospital of China Medical University, Shenyang 110001, China; School and Hospital of Stomatology, China Medical University, Shenyang 110001, China; Liaoning Provincial Key Laboratory of Oral Diseases, Shenyang 110001, China; School and Hospital of Stomatology, China Medical University, Shenyang 110001, China; Liaoning Provincial Key Laboratory of Oral Diseases, Shenyang 110001, China; Department of Plastic Surgery, The First Hospital of China Medical University, Shenyang 110001, China; Department of Plastic Surgery, The First Hospital of China Medical University, Shenyang 110001, China; Liaoning Provincial Key Laboratory of Oral Diseases, Shenyang 110001, China; School and Hospital of Stomatology, China Medical University, Shenyang 110001, China; Liaoning Provincial Key Laboratory of Oral Diseases, Shenyang 110001, China

**Keywords:** adipose-derived stem cells, bone tissue engineering, inflammation, angiogenesis, osteogenesis

## Abstract

In recent decades, bone tissue engineering, which is supported by scaffold, seed cells and bioactive molecules (BMs), has provided new hope and direction for treating bone defects. In terms of seed cells, compared to bone marrow mesenchymal stem cells, which were widely utilized in previous years, adipose-derived stem cells (ADSCs) are becoming increasingly favored by researchers due to their abundant sources, easy availability and multi-differentiation potentials. However, there is no systematic theoretical basis for selecting appropriate biomaterials loaded with ADSCs. In this review, the regulatory effects of various biomaterials on the behavior of ADSCs are summarized from four perspectives, including biocompatibility, inflammation regulation, angiogenesis and osteogenesis, to illustrate the potential of combining various materials with ADSCs for the treatment of bone defects. In addition, we conclude the influence of additional application of various BMs on the bone repair effect of ADSCs, in order to provide more evidences and support for the selection or preparation of suitable biomaterials and BMs to work with ADSCs. More importantly, the associated clinical case reports and experiments are generalized to provide additional ideas for the clinical transformation and application of bone tissue engineering loaded with ADSCs.

## Graphical abstract

**Figure:**
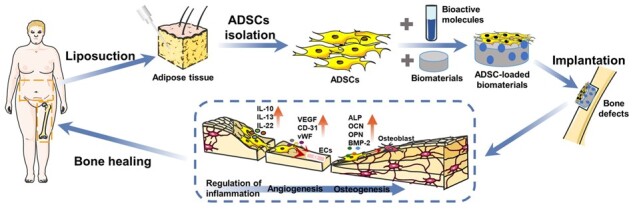

## Introduction

As the incidence of bone injuries caused by sports, trauma and inflammation elevates year by year, the demand for bone replacement or regeneration at the damaged sites increases [1]. Bone has potent regenerative abilities; thus, small bone defects are frequently healed by self-repair. Nonetheless, severe trauma, bone tumor resection and bone infection result in large bone defects (typically >2 cm, depending on the anatomical site [2]) that exceed the bone’s self-healing capacity and present formidable challenges to clinicians [3]. Internal fixation, autologous transplantation and allotransplantation are the primary therapeutic methods for bone repair; however, their application is limited by complications such as immune rejection, chronic pain at the donor site, potential immunogenicity and insufficient donor tissue supply [4]. In recent decades, the tissue engineering platform, in conjunction with materials science and stem cell technology, has been extensively applied to the production of bone substitutes [5].

Bone tissue engineering (BTE) comprises scaffolds, seed cells and bioactive molecules (BMs) [6]. Scaffolds with excellent biocompatibility and mechanical properties support the defect site and offer carriers for cell growth and tissue regeneration. Additionally, BMs deliver appropriate signals to induce osteogenic differentiation in cells. When stimulated by them, seed cells can differentiate directionally and generate sufficient bone tissue at the site of the defect to promote bone healing [6]. Selecting appropriate seed cells is an important part of BTE. Differentiated cells obtained through a biopsy are the preferred source of seed cells for regenerative therapy [7]. Nonetheless, the use of differentiated cells in tissue engineering applications is frequently constrained by the small number of harvested cells and the low proliferation potential when amplified *in vitro*, particularly in the treatment of elderly and multimorbid patients requiring regenerative therapy [8]. The ease of obtaining stem cells, coupled with their superior proliferative capacity, makes them ideal candidates for regenerative therapy [7]. In addition to bone marrow, adipose tissue, cord blood and dental tissue, a variety of tissues contain stem cells used for transplantation [9]. Compared to other tissues, adipose tissue covers a larger area of the organism, making the sampling location easier to access. In addition, the operation causes patients less trauma and pain, and the incidence rate of donor site complications is extremely low [10]. Adipose-derived stem cells (ADSCs), extracted directly from adipose tissue, have been found to possess multi-differentiation potentials (lipogenic, osteoblastic, chondrogenic differentiation, etc.), as well as the ability to self-renew and proliferate *in vitro* [11]. In addition, as non-polarized mesodermal cells, ADSCs are believed to be able to differentiate into osteoblasts (also lacking polarity) with relative ease. These advantages make them better candidates for BTE [7]. In recent years, a growing number of studies have employed ADSCs as BTE seed cells, thus leveraging on their plasticity and versatility. Correspondingly, ADSC-loaded materials have emerged as a new trend for the treatment of bone defects. However, the effects of different biomaterials on the behavior of ADSCs have not been systematically summarized, resulting in a lack of a theoretical basis for selecting the most compatible biomaterials with ADSCs.

The primary objective of this review is to summarize recent advances and applications of ADSC-loaded materials in the process of bone defect repair and bone regeneration. Accordingly, the regulatory effects of various biomaterials on the behavior of ADSCs are summarized from four aspects, including biocompatibility, inflammation regulation, angiogenesis and osteogenesis, to demonstrate the potential of combining various materials and ADSCs for the treatment of bone defects. Notably, BMs, such as growth factors (GFs), as well as ADSCs-exosomes, inorganic ions and small molecule drugs that have been used in combination with ADSCs for BTE strategies were also reviewed. Finally, we summarized the case reports and trials of the clinical application of ADSC-loaded materials over the past 5 years in an effort to provide additional evidences and ideas for the clinical transformation and application of ADSC-loaded materials.

## The elements of BTE

The combination of ideal seed cells, suitable scaffolds and BMs is the key to the success of BTE [12]. Bone tissue is known to be composed of a variety of cells and extracellular matrix (ECM), the latter is mainly composed of organic and inorganic components. By simulating ECM’s constituents, the scaffold in BTE functions as the ECM [2]. Various polymers and bioceramics have been used as potential candidates for simulating the organic (type I collagen, Col I) and inorganic (hydroxyapatite, HAp) components of bone ECM, respectively. Polymers can be synthetic or naturally sourced. Synthetic polymers, such as polycaprolactone (PCL), polylactic acid (PLA), poly (DL-lactide-co-glycolide) (PLGA), etc., are widely used due to their superior biological properties and longer shelf life. On the other hand, due to their inherent biocompatibility and degradability, natural polymers are also regarded as excellent candidates for BTE [13]. Currently, bioceramics with a high biological activity, such as HAp, β-tricalcium phosphate (β-TCP), biphasic calcium phosphate and bioglass (BG), have been widely employed [14, 15]. In addition, the potential of classical metal materials, such as titanium (Ti) and magnesium (Mg) alloys, in conjunction with ADSCs, has been investigated [16, 17]. Although the related research is still in its exploratory phase, the potential for future applications is highly encouraging. Although numerous advantages of various biomaterials as scaffold materials have been demonstrated, they present a significant disadvantage when used alone, thus, exacerbating their application. Therefore, the fabrication of composite scaffolds by combining two or more existing biomaterials is also the primary direction of research in the BTE field. However, these materials cannot replicate the complex microenvironment of natural bone tissue [18]. In this regard, it has been discovered that the tissue-specific natural decellularized ECM (dECM) scaffolds can retain the physicochemical signals and biological properties of ECM after undergoing acellularization [19]. This opens up novel avenues for biomaterials derived from human organs/tissues (O/T) for use in tissue engineering. The application of ADSCs in combination with biomaterials in bone regeneration strategies is described in detail below.

Due to their high proliferative potential and superior osteogenic capability [20], bone marrow mesenchymal stem cells (BMSCs) have been regarded as ideal candidate cells for use as seed cells over the past few decades [21]. Nonetheless, the extraction of BMSCs is a highly invasive procedure accompanied by frequent complications and low cell production. This motivates researchers to explore a more suitable candidate cell [22]. Extensive research and application of regenerative medicine rely heavily on the abundant supply of seed cells [12]. In recent years, researchers have found another stem cell that is easily obtained in adipose tissue, namely ADSCs. They were obtained primarily from liposuction or excised subcutaneous adipose tissues, then digested and centrifuged with collagenase [12]. Compared to bone marrow aspirates, which contain only a small number of stem cells (0.001–0.01% of nucleated cells), fat aspirates contain a large amount of ADSCs, comprising about 1–5% of nucleated cells [23]. In terms of biological function, ADSCs have a similar capacity for bone differentiation to BMSCs [20], but they are less sensitive to aging [24]. In addition, ADSCs have been demonstrated to have unique immunomodulatory effects [25]. Accordingly, ADSCs not only regulate the immune response by promoting the proliferation and function of immune cells but also secrete higher amounts of anti-inflammatory proteins, such as tumor necrosis factor-α (TNF-α), interleukin-4 (IL-6) and IL-1 receptor antagonists [26]. With the discovery of the function of ADSCs to secrete vascular endothelial growth factor (VEGF) and their capacity to differentiate into vascular endothelial cells (ECs), the efficacy of ADSCs in promoting angiogenesis has been gradually investigated [27]. Thus, adipose tissue appears to be a promising substitute for bone marrow as a source of seed cells for BTE [23].

In order to achieve better bone regeneration, the application of BMs may result in a favorable local microenvironment [28]. When GFs released from the scaffold material are long-lasting and stable, the behaviors of cell adhesion, proliferation and differentiation are effectively optimized, and the bone regeneration ability of the implanted scaffold is significantly enhanced [28–30]. Numerous GFs are associated with osteogenesis, including bone morphogenetic protein (BMP), platelet-derived growth factor BB (PDGF-BB) and fibroblast growth factor (FGF). BMP is a critical regulator of cell proliferation and differentiation. Based on the studies related to BTE, BMP-2 comprises the most researched and effective BMP for osteogenesis [31]. PDGF is predominantly isolated from platelets. As a subgroup, PDGF-BB comprises a significant factor regulating angiogenesis and cell proliferation [32]. It appears early in bone defect repair and persists throughout the entire process, accelerating bone resorption and promoting bone remodeling [33]. FGF, plays a role in vital cellular functions, including mitosis and angiogenesis [34]. It was discovered to be involved in every stage of bone formation [35], and is sometimes supplemented in culture media as osteogenic formulations for ADSCs [36]. As researchers delved deeper, they discovered that the osteogenic effects of a cell were related not only to its differentiation capacity but also to its paracrine effects [37]. Correspondingly, exosomes released from ADSCs have been demonstrated to be a new ‘inducer’ that efficiently promotes osteogenesis. Due to its ability to transport various biological components, such as proteins, lipids and nucleic acids, exosomes play a crucial role in cell-to-cell communication. In addition, they regulate cell functions such as proliferation, migration and differentiation. Therefore, as a kind of BMs, exosomes have considerable application potential in BTE [38].

**Figure 1.**
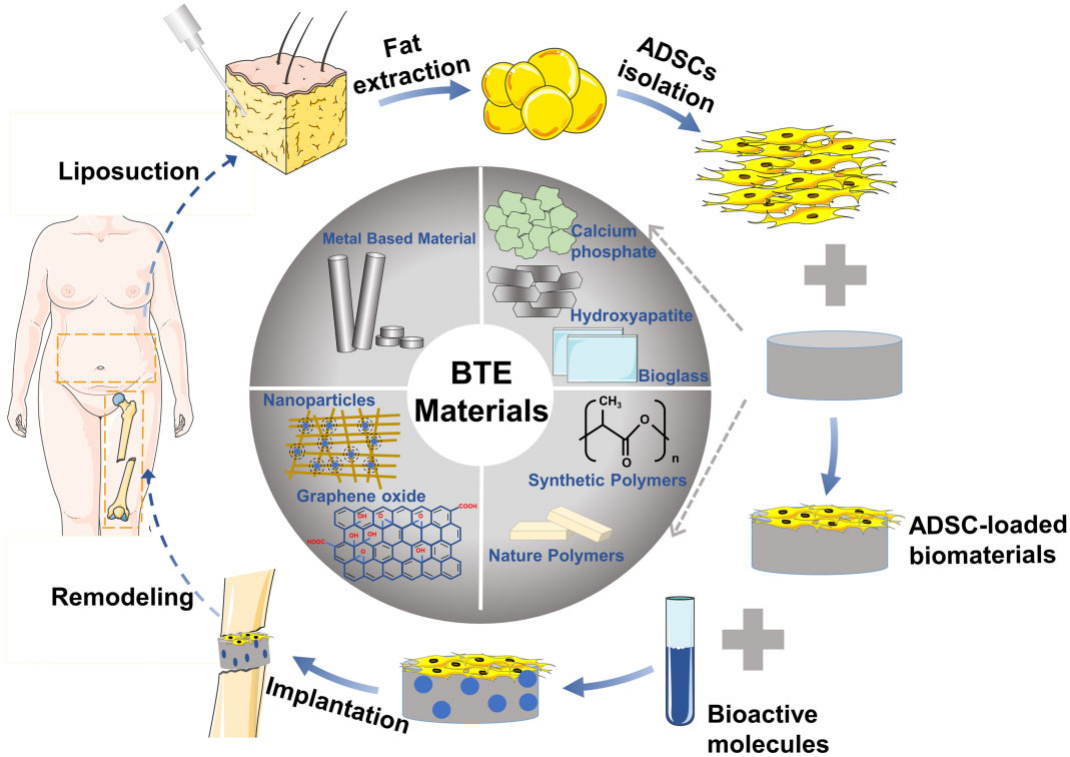
ADSC-loaded approaches in BTE. First, ADSCs are extracted and separated by collagenase digestion from subcutaneous adipose tissue obtained by liposuction or excision. Second, ADSCs are loaded on suitable bioactive materials. BMs are added as biological inducers to help the osteogenic differentiation of ADSCs. Finally, implanting the tissue-engineered bone containing ADSCs into the bone defect site to induce bone tissue regeneration and repair the bone defect. At present, biomaterials used in BTE can be roughly divided into four categories: metallic materials, ceramic materials, polymeric materials, nanoparticles and so on. Various biomaterials have been tried to be combined with ADSCs for BTE and show promising bone repair effects.

A growing number of studies are currently investigating the possibility of combining various biomaterials with ADSCs for BTE. In addition, some studies have attempted to improve bone repair outcomes by supplementing ADSC-loaded scaffolds with BMs. The entire process is summarized in Figure 1. In addition, related content is introduced in detail in the following sections.

## Biocompatibility

Biocompatibility, as redefined in the book, is the ability of a material to perform with an appropriate host response in a specific application [39]. Biocompatibility mainly includes cytotoxicity, allergic reaction, stimulation or intracutaneous reactivity, systemic toxicity, subchronic toxicity, genotoxicity and blood compatibility [40]. We found that the current researches on the biocompatibility of ADSC-loaded materials mainly focused on ‘cytotoxicity’ [41–43]. Therefore, we primarily discuss the cytotoxicity of metallic, ceramic and polymeric biomaterials to ADSCs in this part.

### Metallic biomaterials

Due to their low immunogenicity, corrosion resistance and excellent mechanical properties, Ti and its alloys have been widely used in recent decades as repair therapy for bone regeneration [44, 45]. However, its biological inertia and poor wear resistance often lead to implant failure, and the low compatibility of the scaffold surface with protein or cells will result in infection, inflammation and bone fusion [21]. Therefore, researchers began examining how to enhance the biocompatibility of the ADSC-loaded Ti alloy. After hydrothermal treatment with sodium hydroxide (NaOH) and sulfuric acid (H_2_SO_4_) on the surface of a Ti scaffold, the scaffold had no toxic effect on ADSCs, and the hydrothermal solution on the surface had no appreciable effect on cell viability [21]. The Ti scaffold was treated with potassium hydroxide (KOH) hydrothermal treatment and plasma etching, respectively, and the excellent cell survival rate on the two surfaces demonstrated that neither of the two processing techniques was harmful or stressful to ADSCs [41]. In addition, it was discovered that the surface roughness of the material was essential for the biocompatibility of the cells. ADSCs were seeded onto polished, sandblasted, and machined Ti and zirconia implants, respectively, and it was discovered that the rough surfaces produced superior results. Although Ti implants were more cytotoxic than zirconia implants, they had no effect on cell viability [16]. Overall, the aforementioned methods are on the micro scale. Since the bone tissue is composed of various nano-sized components, people have also studied different nano-surface modification strategies to remarkably improve the biocompatibility [46]. TiO_2_ nanotubes provide a hierarchical template similar to natural bone tissue. The biocompatibility between ADSCs and TiO_2_ nanotubes is affected differently by nanotubes with varying crystalline phases produced at different annealing temperatures. TiO_2_ nanotubes with anatase phase matrix formed by annealing at 530°C and anatase/rutile phase matrix formed by annealing at 630°C not only promoted the proliferation of ADSCs but also improved the cell elongation of ADSCs, optimizing the cell compatibility to a great extent [42]. In another similar study, anatase matrix titanium dioxide nanotubes formed at comparable annealing temperatures were found to promote the optimal combination and interaction between ADSCs and the material surface [43]. Secondly, the compatibility of nanotubes with cells varies based on their diameter. TiO_2_ nanotubes with diameters of 70 and 110 nm were found to promote the proliferation of ADSCs, whereas those with a diameter of 160 nm demonstrated a reduction in activity. This suggests that nanotubes larger than 110 nm may not be compatible with ADSCs [46].

Mg and zinc (Zn), as examples of biodegradable and highly biocompatible metals, have been designed and manufactured for the healing and repair of bone tissue diseases [47–50]. Mg corrodes rapidly *in vivo* in chloride environment, causing unanticipated retardation of its mechanical integrity before new bone tissue is regenerated and remodeled. Moreover, hydrogen gas, which is toxic to the human body, is formed and accumulated in the surrounding tissues if the degradation rate is rapid [51]. In one study, Mg-2Zn-1Ca alloy extracts were diluted at 10%, 50% and 100% concentrations to determine the cytotoxicity of the alloy against ADSCs. Cell proliferation was found to be unaffected by the presence of diluted Mg. Compared to the 10% extracts, the 50% extracts appeared to be the optimal concentration for cell proliferation and survival [49]. Zn is an essential trace element found predominantly in bone tissue. Various *in vitro* and *in vivo* studies have demonstrated that pure zinc has good biocompatibility [52]. However, the safe concentration level of Zn^2+^ depends on the type of cell line, and different cells exhibit varying levels of tolerance. When various concentrations of Zn^2+^ were added to mineralized collagen-based scaffolds, it was discovered that, in terms of ADSCs, 2.5 mg/ml of Zn^2+^ had the highest metabolic activity and the greatest cell proliferation in long-term culture, whereas Zn^2+^, used at a dosage of 5 mg/ml group and 0.5 mg/ml were non-toxic to cells, although with a lower metabolic activity [50]. In addition to the above-mentioned metals and ions, some metal ions with low toxicity, such as strontium ion (Sr^2+^) and rare earth element gadolinium ion (Gd^3+^), have been found to have good biocompatibility with ADSCs [53, 54]. Nevertheless, due to the cytotoxicity of Gd^3+^ at high concentrations, Gd^3+^ should be carefully considered when preparing related scaffolds [54].

### Ceramic biomaterials

Bioceramics with excellent biocompatibility are excellent substitutes for the inorganic components of bone tissues. Different doses of the same kind of materials are very important to the biocompatibility of cells. BGs without P_2_O_5_, composed of SiO_2_, CaO and Na_2_O groups, were found to affect the cell metabolism of ADSCs dose-dependently. ADSCs retained a high level of biocompatibility at concentrations as high as 400 μg/ml; however, once the concentration reached 1000 µg/ml, the cells died [14]. Similarly, three different concentrations of nHAp were added to the polymer scaffold in a study conducted by. The results demonstrated that the survival rate of cells on scaffolds containing 10% nHAp was significantly higher than those containing 5% and 15% [55]. In another study, the respective concentrations of HAp were 1%, 5% and 10%. The cells cultured under three conditions all survived, and the percentage of dead cells was <1.6%, indicating that 10% HAp might be more suitable for culturing ADSCs [56]. The surface morphology is also one of the primary determinants of the interaction between materials and cells. ADSCs can survive on all three HAp surfaces (honeycomb, pillars and isolated islands) produced by a micro-casting technique; however, cell proliferation is significantly higher on pillars and isolated islands than on honeycomb, and the uneven distribution of paxillin may be one of the reasons for the decrease in cell adhesion and diffusion on honeycomb substrates [57]. Additionally, due to the delay of biodegradability, the prolonged residence time of HAp *in vivo* is likely to impede bone remodeling. β-TCP, on the other hand, is completely degraded and does not remain in the tissue after bone formation, thereby avoiding the infection and inflammation issues that can arise from long-term implantation. It has also been reported that it is highly biocompatible with ADSCs [58]. In addition, although scaffolds containing high, medium and low doses of β-TCP were not toxic to cells, scaffolds containing a high dose of β-TCP (triple β-TCP) demonstrated a greater cell viability and biocompatibility [59].

### Polymeric biomaterials

As natural polymers, chitosan has been widely used for bone regeneration therapy due to their good biocompatibility. ADSCs adhered to the surface of chitosan scaffolds with varying porosity (10%, 15% and 20%) all demonstrated excellent biocompatibility with the material [60]. For a polymer to be utilized in bone tissue regeneration, its structure needs to be modified to some extent. Because such type of reconstruction is challenging for natural polymers, synthetic polymers are used as inexpensive supplementary biomaterials to achieve good compatibility and mechanical strength. In this regard, different concentrations of PCL (0.5, 0.05, 0.005 mg/ml) were shown to be non-toxic to ADSCs and to have no negative effects on the activity of ADSCs [61]. In addition, PCL with a low molecular weight (40 000 number average molecular weight) has been shown to have better biocompatibility [62]. However, the hydrophobicity of PCL inhibits cell adhesion, thereby impeding cell proliferation and diminishing its biocompatibility. Some bioceramics, such as HAp and BGs, have been shown to significantly improve the biological characteristics of PCL-based scaffolds [24, 63]. However, the PCL scaffold containing 20 wt.% borate bioactive glass (13-93b3 or b3) was found to increase ADSC mortality [64]. One possible explanation is that the ion release and pH shift caused by the dissolution of B3 glass cause cell damage [64]. Therefore, the addition and proportion of BG must be investigated further. Moreover, compared to the 3D-printed PCL+b3 BG scaffold, the scaffold with near-field electrospinning technology has less toxicity to ADSCs, greater cell proliferation and improved biocompatibility [64]. In addition, the dual-scale anisotropic PCL scaffold fabricated by a screw-assisted additive manufacturing technique combined with rotational electrospinning was non-cytotoxic to ADSCs and had a high seeding efficiency and ADSCs’ proliferation ability [65]. The use of graphene in the development and production of biomaterials has been the subject of heated debate. When the poly(epsilon-caprolactone) scaffold was modified with small doses of original graphene (0.13 wt.%, 0.50 wt.% and 0.78 wt.%) as a coating, the cell viability and proliferation rate increased with increasing graphene concentration, and no immune/inflammatory reaction was observed *in vitro* [66]. Variously, high doses of graphene oxide (GO) (2 wt.% and 3 wt.%) were added to chitosan-based 3D Scaffold, ADSCs exhibited statistically significant higher vitality than those exposed to low GO content (0.5 wt.% and 1 wt.%) [67]. This demonstrates that the biocompatibility between GO and ADSCs depends partly on the concentration of GO. With the increasing use of nanoparticles in the field of bone regeneration, researchers have paid particular attention to high-performance nanoparticles. Various reports show that the size, concentration, surface properties, shape and structure of nanoparticles are closely related to their biocompatibility or toxicity [63, 64]. After different concentrations of gold nanoparticles (GNPs) (0, 50 and 200 μM) were attached to a PLGA sheet (PLGA-SH), the results showed that GNPs increased the activity of human ADSCs (hADSCs) in a dose-dependent manner within 48 h [68]. In another study, a similar conclusion was reached, except that after 7 days, the cell viability of high concentration of GNPs (200 μM) began to decline, and the effect was not as effective as that of low concentration GNPs [69]. In nature, iron oxide nanoparticles (IONPs) exist in numerous forms, including magnetite (Fe_3_O_4_), hematite (α-Fe_2_O_3_) and maghemite (γ-Fe_2_O_3_). The concentration of IONPs is the key factor on cell viability when incubated with cells. Low iron concentration (50 and 100 mg/ml IONPs) had no obvious toxic effect on the proliferation of rADSCs, whereas the cell proliferation ability decreased at 200 mg/ml [70].

## Regulation of inflammatory effect

Inflammation is the first stage of bone healing after bone injury [71]. During the acute phase (0–7 days after bone defect), macrophages are recruited and rapidly infiltrate the defect area, most of which are M1 macrophages. During the subacute phase (7–28 days), M1 inflammatory phenotype macrophages gradually switch to M2 anti-inflammatory phenotype, which has been reported to promote bone regeneration [72, 73]. Nonetheless, the long-term presence of M1 macrophages often leads to the excessive secretion of pro-inflammatory factors, which triggers chronic inflammation, delayed wound healing, and even scaffold rejection and additional tissue damage [74]. Therefore, in the early stage after bone injury, timely resolution of inflammation is critical to promote bone regeneration.

When injecting ADSCs into 3D-printed PCL/TCP scaffolds and fixing them in the maxillary defect of beagles, only a certain degree of inflammation existed prior to scaffold implantation was observed, and upon histopathologic examination, no severe immune injection was observed [25]. Similarly, no adverse inflammatory tissue reactions were observed when the μRB-based hydrogel was injected into an immunocompetent mouse cranial defect model [73]. Why can biomaterials loaded with ADSCs resolve inflammation in a timely manner? On the one hand, the anti-inflammatory properties of biomaterials themselves exert a large significant effect [75]. For instance, scholars applied the co-culture system of ADSCs and amniotic membrane (AM) in a rat periodontal defect model, and achieved excellent anti-inflammatory properties, which was mainly attributed to the strong anti-inflammatory and tissue repair effects of AM [75]. On the other hand, ADSCs themselves exhibit excellent immunomodulatory properties. Studies found that compared with BMSCs, ADSCs, as a more stable and controllable source of stem cells, presenting greater advantages in regulating inflammation [76]. ADSCs can inhibit the production of pro-inflammatory cytokines, including interferon (IFN-γ), TNF-α, IL-1α, IL-1β, IL-6, IL-12, IL-15 and IL-17, and increase the level of anti-inflammatory IL-10 [26]. A study showed that the recombinant human BMP-2 (rhBMP-2)-enriched PLGA scaffolds combined with hADSCs reduced rhBMP-2-induced inflammatory responses. The group treated with hADSCs yielded the smallest number of inflammatory foci and giant cells [77]. After treated with hADSCs, various inflammatory mediators were inhibited, and antigen-specific Treg cells with the capacity to suppress collagen-specific T-cell responses were induced [77]. Moreover, selecting appropriate materials will further regulate the secretion of inflammatory factors by ADSCs. When cultured on porous HAp/Col-derived Bio-Oss^®^\Avitene scaffolds, hADSCs were found that reduced the expression of several pro-inflammatory chemokines, such as CXCL1, CXCL8 and the upregulated expression of anti-inflammatory cytokines, such as IL-13 and IL-22 [78]. The immune regulation behavior of ADSCs is fully exerted under the action of Bio-Oss^®^\Avitene scaffolds, which eventually leads to better osteogenesis effect [78]. Timely construction of an immune microenvironment conducive to osteogenesis is the key to the successful bone regeneration of ADSC-loaded scaffolds [79]. Up to now, there are still few studies on the effects of various biomaterials on ADSCs to release inflammatory factors, thus regulating inflammatory response. For the future research, it is very important to make more BTE scaffolds with immunomodulatory potential, which can act on ADSCs to release inflammatory factors, thus regulating inflammatory response related to bone tissue repair.

## Angiogenic effect

The growth of bone tissue is primarily dependent on the branches of the great vessels, and 10–15% of cardiac output is reportedly allocated to the skeletal system [80]. Therefore, for highly vascularized bone tissue, neovascularization is a necessary condition for bone defect repair, as well as an indispensable process for creating a new vascular network to increase the oxygen and nutrients required for regeneration [81]. Nevertheless, neovascularization has always been a significant challenge. ADSCs, as highly efficient multi-directional stem cells, can not only differentiate into chondrocytes and osteoblasts [82], but it has also been demonstrated that they can differentiate into ECs [83]. Concurrently, they can paracrine cytokines such as VEGF to stimulate angiogenesis [84]. In order to explore the roles of ADSCs in angiogenesis when they are combined with biomaterials applied to BTE, scientists have investigated multiple aspects, including pretreatment of ADSCs, combination with various biomaterials with excellent mechanical properties, and the addition of BMs. The following section describes the roles of ADSCs in angiogenesis from the aforementioned perspectives (Table 1).

**Table 1.** Angiogenesis of ADSC-loaded materials

Biomaterials	Cell	Conduction	Animals	Angiogenesis-Related Change	Ref.
PLC/Col/HA	hADSCs+HUVEC	DMEM+EGM-2	NA	Formation of capillaries (+)	[87]
PCL/heparin-gelatin	hADSCs+HUVEC	DMEM+EGM-2	NA	Expression of CD-31 (+)	[88]
Ti implants	ADSCs+HUVEC	NA	Femur and subcutaneous model in rabbits	Vasculogenic network formation; Expression of SDF-1α, CD-34, vWF (+); Cell migration (+)	[89]
Silk-BG	hADSCs+pECs	NA	Distal epiphyseal region of femurs in rabbits	ECs migration (+); Expression of ANGPTN1, eNOS, CXCR4, CD-31(+)	[86]
DCS-CHA	rADSCs	OM, EC medium	Mice at subcutaneous tissue	Expression of CD-31 (+); Blood vessel count (+)	[83]
DCS-PLL-CHA	rADSCs	OM, EC medium	Radius bone defect model in rabbits	Expression of VEGF, CD31 (+)	[82]
Col/HAp	hADSCs	OM	Athymic nude mice at the cervical subcutaneous tissue	Expression of VEGF (+); Blood vessel count (+)	[84]
PPX-BM	hADSCs	NA	Calvarial defects in rat	Formation of capillaries (+)	[30]
52S-BG	hADSCs	NA	Zebrafish embryos	Expression of VEGF (+); Vascular loops count (+); SIV bud length (+)	[14]
WH-C	VEGF-ADSCs	OM	Calvarial defect in mouse	Expression of VEGF (+); Blood vessel count (+)	[100]
Sr-GO-Col	hADSCs+HUVEC	NA	Calvarial defect	Expression of VEGF, PDGF-BB, CD-31 (+); ECs migration (+); Numbers of junctions increased	[97]
Col/HA	hADSCs+HUVEC	DMEM+F-12; LIPUS	SD rats at subcutaneous sites	Expression of CD-31, VE-cadherin (+); Blood vessel count (+)	[105]

OM,osteogenic medium; ‘(+)’ represents upregulated or promoted; NA, not applicable.

### Pretreatment of ADSCs

**Figure 2.**
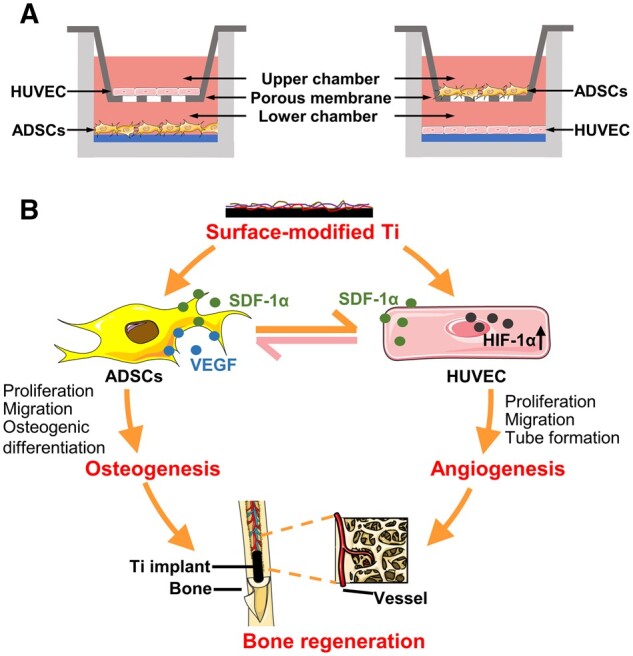
(**A**) Schematic illustration of ADSCs and HUVEC co-culture system. Surface-modified Ti implants directly promote the adhesion, proliferation, migration and differentiation of ADSCs and HUVECs. (**B**) Schematic illustration of coupled osteogenesis and angiogenesis by surface-modified Ti substrates for enhanced bone healing. Angiogenic factors VEGF and SDF-1α secreted by ADSCs act on HUVECs and promote angiogenic effect. In turn, HUVECs promoted the migration of ADSCs through paracrine action.

Studies have demonstrated that angiogenesis and osteogenesis are mutually coupled in the process of bone formation and remodeling [85]. Correspondingly, *in vitro* and *in vivo* studies have demonstrated that scaffold materials containing ADSCs could trigger stronger angiogenesis, a necessary condition for bone defect repair [86]. In a study conducted by in 2018, the manufactured PLC/Col/HA scaffold promoted the adhesion of human umbilical vascular endothelial cells (HUVECs); however, no vascular network was formed. Conversely, it was evident that capillary formation occurred on scaffolds co-cultured with ADSCs [87]. Similarly, when HUVECs were co-cultured with ADSCs, the expression of the endothelial marker CD-31 increased, and the formation of ECs monolayer achieved proper endothelialization [88]. Under co-culture conditions, numerous cytokines secreted by ADSCs promote normal growth and formation of mature endothelium, which may account for this dramatic change in HUVECs [87, 88]. What is the precise mechanism by which ADSCs and ECs co-culture promotes angiogenesis? In 2018, two studies have already provided an answer to this question. Specifically, the expression of SDF-1α and VEGF, which were secreted by ADSCs in a paracrine fashion, increased on the multilayer film-coated titanium substrates, and the interaction between the chemokine SDF-1/CXCR4 axis and VEGF/VEGF receptor axis further promoted the migration and revascularization of HUVECs. In turn, HUVECs enhanced the migration of ADSCs through its paracrine action (Figure 2) [89]. Similarly, ECs and ADSCs seeded on silk-bioactive glass reinforced scaffolds increased the expression of CXCR4, which was followed by the formation of the plexus-like primitive network, the homing of primary ECs and their migration to differentiated hADSCs expressing CXCR4 [86]. Thus, it is evident that the SDF-1/CXCR4 axis is a crucial signaling pathway for angiogenesis that has been discovered and studied thus far, as well as a crucial future research direction for vascular signaling pathways.

In conventional tissue engineering, seed cells and biomaterials are combined to treat large bone defects. Nonetheless, when cells are digested with proteolytic enzymes such as trypsin, a significant proportion may lose their capacity to differentiate [90]. Moreover, in some instances, the structure of the injured part cannot support the inoculation of cell suspension, making it difficult to control the location and quantity of the injected cells [90]. Cell sheet (CS) engineering, a modern biological technique for collecting cells from the bottom of a culture dish by physical or mechanical means, not only completely preserves the ECM and surface proteins but also prevents cell loss and improves utilization, preserving the entire signal communication network and promoting cell adhesion to frameworks [91]. CS engineering has made significant advancements over the past few years. Accordingly, double-cell sheets (DCS) consisting of the osteogenic CS and EC sheet, which were cultured in OM and EC medium, and obtained by directional differentiation of ADSCs, have been demonstrated to have osteogenic and angiogenic properties [83]. Subsequently, researchers coated DCS on the surface of coral HAp (CHA) scaffold, surprisingly found that when the EC sheet was inside and the osteogenic CS was outside, the angiogenesis capacity of the complex (DCS-CHA) was optimal, as was the mineralization capacity of collagen fibers. The combination of two CSs enabled DCS-CHA to have an exceptional osteogenic potential [83]. The reason may be that the ECs carried by the EC sheet formed a vascular network at the center of the graft, and the outer osteogenic sheet formed an efficient signal and material transmission pathway with the surrounding tissues [83]. After connecting the internal and external blood vessels, the graft quickly restored the blood supply. In addition, it was discovered that the pores in CHA scaffolds facilitate cell migration and adhesion, thereby forming a more stable vascular network [83]. In order to study *in vivo* rabbit radius defect repair, researchers added polylysine (PLL), a laminin that promotes the adhesion and proliferation of ADSCs, to DCS-CHA [82]. They discovered that DCS composite could temporarily act as a biological barrier at the initial stage, effectively preventing fibrous tissue from growing directly into CHA pores, thus affecting the ossification process of the entire defect sites. At 8 weeks post-surgery, a stable blood supply had been established, and there were numerous osteoblasts at the edge of the new bone. At 12 weeks, the majority of the scaffold surface was covered by a fibrous callus [82]. As can be seen, the vascularized tissue-engineered bone constructed by CSs has a high potential for bone regeneration and bone reconstruction, and there is ample room for future development [82]. However, there are still issues to be resolved in the current construction and research of CS engineering. Multilayer CSs are prone to provide insufficient oxygen and nutrients to the internal cells, resulting in cell death. Consequently, the number of CSs that can be layered without causing core ischemia or hypoxia is limited [90]. Thus, future research should focus heavily on the CS technology.

### Different biomaterials

Scaffolds play a major role in tissue engineering, they are used as carriers for the transportation, enrichment and maintenance of ADSCs. The angiogenic properties of ADSCs have been demonstrated, but the exact effects of ADSCs on cell adhesion, migration and angiogenesis when combined with scaffolds composed of different structures and components have not been summarized [84]. 3D-printed scaffolds can be customized with specific porosity, pore size and pore connectivity to optimize cell adhesion, migration and nutrient transport [92]. The 3D porous scaffold poly-p-xylylene (PPX) was implanted in a rat calvarial defect model, and its porous structure greatly promoted the adhesion of hADSCs by providing a highly biocompatible cell channel [30]. Concurrently, the abundant blood microenvironment provided adequate nutrition for osteogenesis, as evidenced by the discovery of massive bone deposition in the central region of the rat calvarial cavity. Particularly, hADSCs are not defined here as cells that directly form new tissues; conversely, they are viewed as ‘medicinal cell factories’ that secrete various BMs in response to the microenvironment and by recruiting endothelial progenitor cells to promote angiogenesis [30]. Scaffold materials influence ADSCs to exert a pro-angiogenic effect, which is not only related to the structure of materials but also has a substantial relationship with material composition. The combination of Col and HAp is regarded as a viable option for simulating the ECM components of BTE [93]. Accordingly, Col/HAp scaffolds that were seeded with hADSCs were implanted into the back of athymic mice [84]. When compared to pure Col scaffold, the Col/HAp scaffolds supported mineral deposition and vascular invasion at comparable rates. The scaffold’s composition could differentially guide vascularized bone regeneration in the ectopic model [84]. The traditional BG used for BTE consists primarily of silicon dioxide (SiO_2_), boron trioxide (B_2_O_3_) or/and phosphorous [94]. Recently, scientists have discovered that BG without phosphorous has a substantial advantage in promoting angiogenesis. A novel BG (52S-BG) with 52% SiO_2_, not containing P_2_O_5_ was discovered to enhance the angiogenesis and osteogenic properties of ADSCs in a dose-dependent manner [14]. Accordingly, a high concentration of 52S-BG (300 μg/ml) significantly increased the expression of the vascular marker (VEGF) and the osteogenic marker (ALP). Furthermore, the application of 52S-BG to zebrafish embryo model *in vivo* further demonstrated its angiogenesis capability [14]. Considering BG’s inherent brittleness and low fracture toughness, they used PCL as the substrate and incorporated 10% 52S-BG to create composite materials that exhibited exceptional angiogenesis capability. In addition, massive calcium phosphate deposition on the surface of the scaffold revealed excellent osteogenic properties [14]. Experts hypothesize that this angiogenic property is related to the release of Si^4+^ from scaffolds; the angiogenic potential of silicate-based BGs has been summarized in detail in the review [95].

### BMs supplement

Incorporating BMs into the regulation of molecular signals is also a method for promoting angiogenesis and bone regeneration [96, 97]. As we all know, VEGF is a crucial factor in the regulation of angiogenesis, as it can promote EC migration, proliferation and vascular invasion [98], and regulate the behavior of osteoblasts [99]. However, its biological half-life is short when it exists as a bioactive factor on the surface of the scaffold. In order to improve the efficiency of gene delivery, researchers modified ADSCs with VEGF using lentiviral gene delivery [100]. This is also a pretreatment of ADSCs by integrating the target gene into the seed cells to increase the efficacy of gene delivery and further prolong the gene expression time of VEGF [101]. The results demonstrated that VEGF-ADSCs, as a type of therapeutic cell, had a significant angiogenic effect, with VEGF secretion increasing with cell growth. However, controlled release is regarded as the key to optimizing the therapeutic effects of GFs [96]. Several studies have pointed out that VEGF has a specific binding region that binds specifically to heparin [102]. Thus, when VEGF-ADSCs were seeded onto heparin-containing whitlockite (WH) scaffolds, the release of VEGF was slow, but continuous and gradually increased, creating an environment rich in VEGF [96]. In this instance, sufficient bioactive factors prompted ADSCs to migrate to the site of the bone defect, where they participated in intramembranous ossification [96]. In addition, the mineralization environment produced by WH promoted the transformation of ADSCs into osteoblasts [100]. In contrast, therapeutic inorganic ions, such as Mg^2+^, Sr^2+^ and Cu^2+^, can be selectively added to the scaffold due to the multiple difficulties of cytokines in decomposition, their lack of specificity or sensitivity to microenvironmental conditions, as well as their higher cost [97]. They are preferred not only because they can stimulate osteogenesis and angiogenesis but also because they are resistant to decomposition and have low microenvironmental specificity or sensitivity [97]. Sr–GO-modified Col scaffold activated the MAPK signaling pathway due to the presence of Sr and GO, resulting in a significant increase in the secretion of VEGF and BMP-2 by ADSCs [97]. When the scaffold was applied to a calvarial defect model *in vivo*, the results demonstrated the best bone regeneration and angiogenesis, and the addition of Sr^2+^ provided a superior microenvironment for more effective bone regeneration [97]. Furthermore, as a promising bone induction factor in regenerative medicine, when the exosomes derived from hADSCs (hADSCs-exos) were densely and uniformly deposited on the PLGA-based scaffold, they could effectively promote the migration and angiogenesis of HUVECs, provide a good blood supply for bone regeneration and accelerate bone regeneration [103].

### Physical treatment

In addition to the aforementioned methods, scientists have discovered that adding auxiliary *in vitro* therapy to tissue engineering therapy can speed up the bone healing process [104]. Low-intensity pulsed ultrasound (LIPUS), a physical treatment, can enhance and regulate the expression of VEGF during the initial phase of bone healing [105]. Previous research primarily centered on chondrocyte culture and endochondral ossification [106]. However, a researcher co-cultured ADSCs and HUVECs on a Col/HA scaffold to examine the effect of LIPUS on angiogenesis [104]. It was observed that on the scaffold received LIPUS therapy, the expression of EC markers significantly increased. Thus, LIPUS played a significant role in the middle and long-term progression of angiogenesis [104]. However, the effect of LIPUS on bone regeneration has not yet been elucidated, which can serve as a basis for future investigation.

## Osteogenic effect

**Figure 3.**
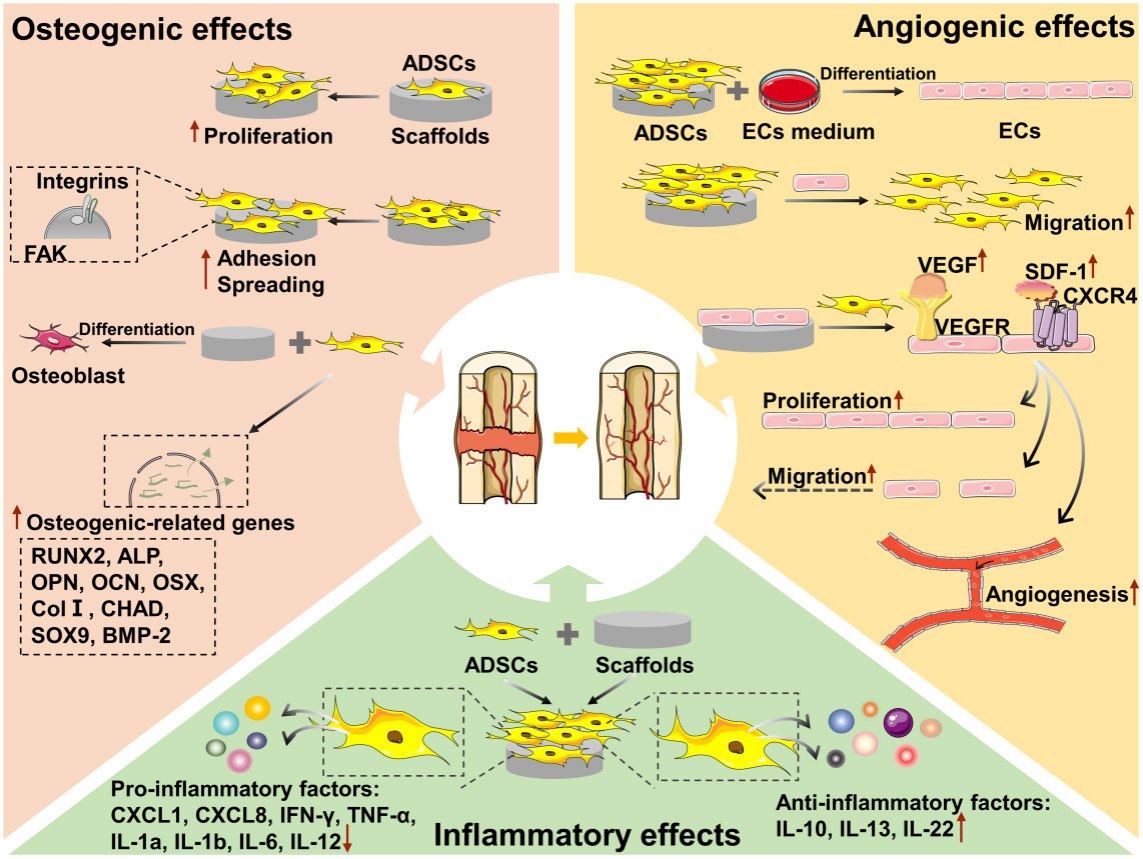
The roles of ADSCs combined with biomaterials in bone regeneration. Under the induction of suitable biomaterials, ADSCs regulate the inflammatory reaction after implantation by upregulating the expression of anti-inflammatory factors, while downregulating the expression of pro-inflammatory factors. More importantly, ADSCs secrete angiogenic factors which promote the angiogenic effects of ECs. In turn, ECs promote the biological behavior of ADSCs through paracrine action, thus achieving angio-osteogenesis coupling. ADSCs also can adhere, spread and proliferate on the surface of biomaterials through the interaction with biomaterials, and differentiate into osteogenic lineage, finally differentiated into bone cells to achieve bone tissue regeneration.

The combination of ADSCs with various biomaterials has been widely tried in BTE. Correspondingly, the osteogenic effects have been studied and compared by examining the effects of different materials on the adhesion, diffusion, proliferation and osteogenic differentiation of ADSCs [15, 16, 107]. In this section, the research status of different materials combined with ADSCs and applied to BTE is presented in detail (Table 2). Furthermore, the influence of different material properties on ADSCs is mentioned in the discussion of the combination of ADSCs and biomaterials (Figure 3).

**Table 2.** Osteogenesis of ADSC-loaded materials

Biomaterials	Cell	Animal	Osteogenesis-related change	Ref.
Mg-containing mesoporous TiO_2_ coatings	hADSCs	NA	Cell viability (+); Adhesion and spreading (+); ALP activity (+); Expression of OPN (+)	[108]
Ti hydrothermally treated with NaOH	hADSCs	NA	Proliferation (+); Adhesion and spreading (+); eposition and expression of OCN (+); Mineralization (+)	[21]
BG-TiNT coating	hADSCs	NA	Proliferation (+); Adhesion and spreading (+); ALP activity (+); Expression of OCN (+); Mineralization (+)	[111]
52S-BG particles	hADSCs	NA	Proliferation (+); ALP activity (+); Expression of RUNX2, ALP, OPN and Col I (+); Mineralization (+)	[14]
HAp-functionalized SF scaffolds	hADSCs	Mouse calvarial bone defect	*In vitro*: Expression of OPN (+); *In vivo*: Formation of mineralized bone (+); Collagen deposition (+)	[15]
Col/HAp Scaffolds	hADSCs	NA	Gene expression of integrins, collagens and laminins (+); Activation of FAK protein (+); Expression of OPN, ALP, ON, CHAD, OSX and SOX9 (+)	[113]
IS-HAp scaffolds	hADSCs	NA	Proliferation (+); Cytoskeletal organization and expression of focal adhesion molecule (+); ALP activity (+); Expression of Col I, RUNX2, ALP, OPN and BMP-2 (+)	[57]
3D printing PCL scaffolds	hADSCs	NA	Proliferation (+); Adhesion and spreading (+); ALP activity (+)	[62]
PCL/Col I/MNPs scaffold	rADSCs	NA	Proliferation (+); Adhesion and spreading (+); ALP activity (+); Expression of Col I, OCN, RUNX2 and BMP-2 (+); Calcium deposition (+)	[24]
Porous PLGA scaffold	hADSCs	Rat calvarial bone defect	Formation of new bone: PLGA scaffold + hADSCs> hADSCs or PLGA scaffold>control	[126]
Fragmented PCL nanofiber/HA hydrogel	ADSCs	NA	Proliferation (+); Adhesion and spreading (+); Expression of Col I, ALP and RUNX2 (+); Mineralization (+)	[136]
γ-Fe_2_O_3_/PLGA/PCL/β-TCP	rADSCs	Rat calvarial defect	ALP activity (+); Expression of RUNX2, BMP-2, Col I and ALP (+); New bone regeneration *in vivo*	[70]
Fe–cat NPs	hADSCs	Back of the nude mice; Rat femur defect	Expression of BMP-2, OPN and OCN (+); Immunofluorescence intensity of OCN (+); Calcium deposition level (+); Immunofluorescence intensity of OCN in nude mice (+); New bone regeneration in rats	[163]
Fe_3_O_4_ NPs/PCL/Col I scaffold	rADSCs	NA	Adhesion and spreading (+); Expression of Col I, Runx2, OCN, ON, and BMP2 (+); Calcium deposition (+)	[24]
α-Fe_2_O_3_/PLGA/PCL scaffolds	rADSCs	NA	Expression of ALP, RUNX2, Col I and OCN (+); Calcium deposition (+)	[128]
Ma-dECM/alginate bioink	hADSCs	NA	Proliferation (+); ALP activity (+); Mineralization (+) Expression of ALP, BMP-2, OCN, OPN (+)	[146]
aoPCL	hADSCs	NA	Calcium deposition (+); Expression of Col 1A1, Col 9A1, OPN, ALPL (+)	[149]

‘(+)’ represents upregulated or promoted; NA, not applicable.

### Metallic biomaterials

**Figure 4.**
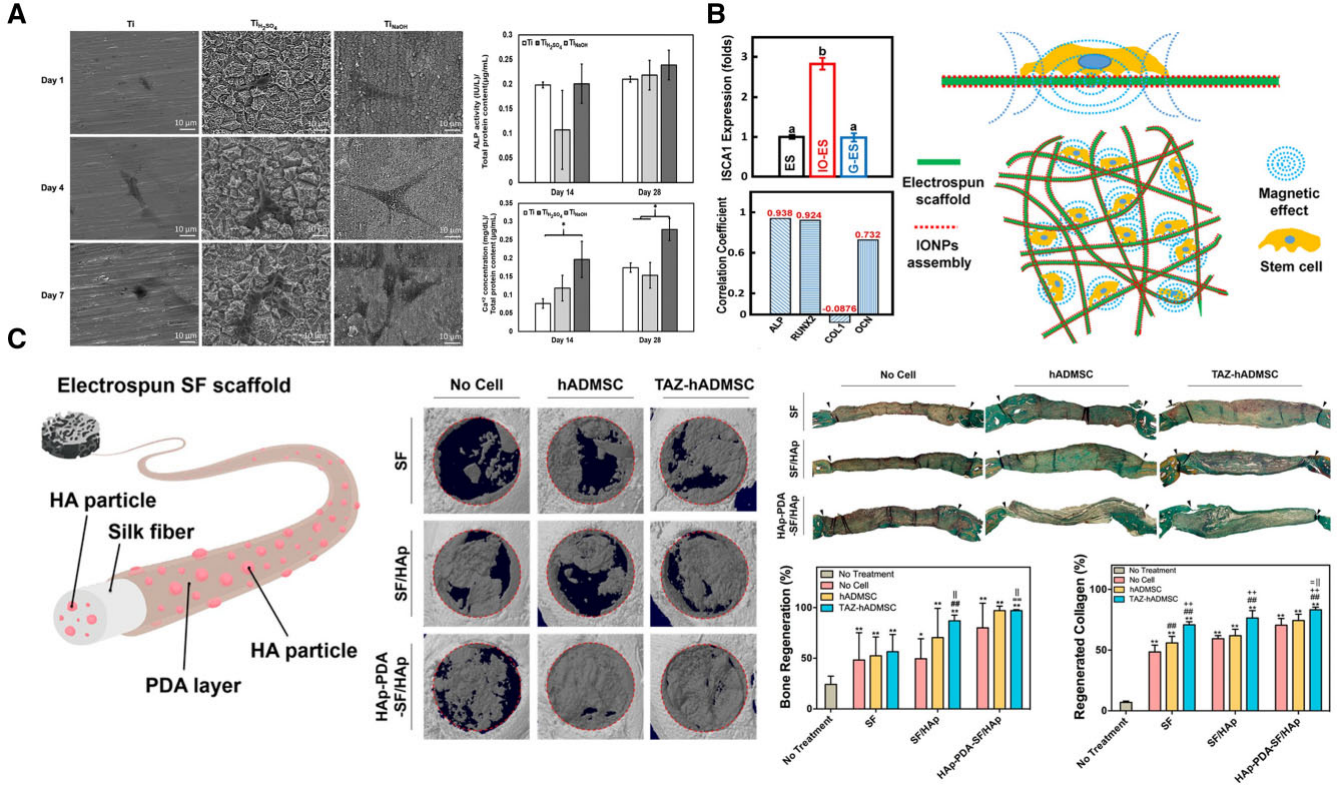
(**A**) SEM images of a higher cell spreading on TiNaOH surfaces. ALP activity and calcium deposition by ADSCs on different surfaces were quantified and normalized. (Reprinted with permission from Ref. [20]. Copyright (2021) American Chemical Society.) (**B**) Schematic showing of the mechanism for effect of the IONPs-assembled electrospun scaffold on the cells. (Reprinted with permission from Ref. [108]. Copyright (2018) American Chemical Society.) (**C**) Schematic illustration of the SF scaffold engineered with two-stage HAp functionalization. Enhanced mineralized bone formation and collagen deposition in critical-sized calvarial bone defects. (Reprinted with permission from Ref. [15]. Copyright (2018) American Chemical Society.).

In a few studies, the osteogenic response of ADSCs to modified Ti scaffold was investigated, indicating the possibility of the two for the treatment of BTE [16, 21, 107–109]. ADSCs were compared with human osteoblasts and human gingival fibroblasts, which can also be used as edaphic cells, on surface-modified Ti materials [16]. Although hADSCs exhibited delayed osteogenic gene expression, indicating a delayed onset of osteogenic differentiation, compared to the osteogenic responses of human osteoblasts and human gingival fibroblasts, hADSCs on the surface-modified Ti scaffold displayed more pronounced osteogenic effects [16]. Moreover, in the absence of osteo-inductive factors, hADSCs exhibited a higher expression of RUNX2, OPN and OCN on sandblasted and acid-etched rough Ti (Ti-SLA) implant surfaces than on smooth polished Ti surfaces [16]. In this regard, the combination of surface-modified Ti scaffold with ADSCs has been highly promising. Regarding the properties of the Ti surface, the formation of relatively rough topography, and in particular the formation of biomimetic nano-topography, is advantageous for the adhesion, diffusion and directional migration of ADSCs, as has been confirmed for a long time [110]. In addition, the difference in nanostructure (differing protrusion sizes) influences the propensity of ADSCs to differentiate into osteogenic or adipogenic phenotypes [110]. Accordingly, several studies have been devoted to the design of nano-topography on the surface of Ti and its alloys to promote the proliferation, adhesion and osteogenic differentiation of ADSCs [21, 107, 108]. In particular, the modifications of Ti alloy surfaces with TiO_2_ or γFe_2_O_3_ nanoparticles were found to be advantageous for the adhesion, proliferation, osteogenic differentiation and extracellular calcium deposition of ADSCs [107]. It is possible that the formation of nano-morphology and the distribution of negative charges are the primary reasons why these modified surfaces promote the biological reaction of ADSCs [107]. Moreover, it has been demonstrated that nanoporous Ti surfaces that resemble bone ECM structure significantly enhance the adhesion, proliferation, diffusion and osteogenic differentiation of ADSCs [21, 108]. However, it should be noted that nano-surface structures that are too uneven, such as high microscale pyramidical structures or deep nanoscale pits, do not promote the survival and osteogenic differentiation of ADSCs [21]. The 2D-like planar surfaces with nano-porous structures treated with sodium hydroxide (TiNaOH surfaces) are more advantageous to bone repair due to their significantly improved interaction with ADSCs (Figure 4A) [21]. Moreover, nanowire structure and hydroxyapatite deposition on the surface of 3D printed porous Ti alloy obtained by hydrothermal treatment significantly enhanced the spreading and elongation of ADSCs, accompanied by the high expression of paxillin, a key regulator of cell adhesion and migration at the edge of the cells [109]. Intriguingly, to further improve the osteogenic capability of the Ti surface, nano-topography was combined with bioactive coating [111]. The Ti surface formed by depositing manganese-containing BG nanoparticles on TiO_2_ nanotube arrays (BG-TiNT) induced osteocalcin (OCN) expression and extracellular calcium deposition in ADSCs [111]. This provides a promising method of Ti surface modification that can combine and utilize various osteogenic promotion methods to a greater extent.

### Ceramic biomaterials

Recently, the feasibility of using ceramic biomaterials as carriers of ADSCs for bone defect repair has been progressively confirmed. Novel 52S-BG particles have been confirmed to possess the ability to stimulate osteogenesis in ADSCs in a concentration-dependent manner [14]. Correspondingly, the ALP activity of ADSCs significantly increased after 14 days in a medium containing 100–300 μg/ml of 52S-BG, but decreased when the concentration was increased to 400 μg/ml [14]. This will guide the combined application of ADSCs and scaffolds containing BGs, as it is essential to control the proportion of BGs or the degradation rate of scaffolds. Considering the promoting effect of BGs on the osteogenesis of ADSCs, BGs were added to ADSC-loaded scaffolds made of other materials to improve the bone repair capability [14, 111, 112]. Relevant contents are mentioned in the description of the relevant materials. BGs, whether used as a coating material or a composite material, provide good osteogenic capability for scaffolds in general.

HAp is an excellent option for creating implants in which ADSCs are transported to bone defect sites. However, due to its inadequate mechanical properties, a number of studies have attempted to create HAp-contained hybrid scaffolds with improved properties [15, 23, 113, 114]. It has been demonstrated that HAp-functionalized electrospun silk fibroin (SF) scaffolds significantly promote ADSCs osteogenic differentiation. In a mouse calvarial bone defect model, transferring hADSCs via HAp-functionalized SF scaffolds resulted in superior bone defect repair compared to using scaffolds or hADSCs alone (Figure 4C) [15]. Moreover, it was discovered that a Col/HAp hybrid scaffold augments the upregulation of integrins, collagens and laminins genes in ADSCs. The upregulation of specific genes associated with bone mineralization and osteogenesis, including OPN, ALP, osteonectin (ON), chondroadherin (CHAD), OSX and transcription factor sex-determining region Y-box 9 (SOX9), further confirmed that the scaffolds promoted the osteogenic differentiation of ADSCs [113]. ADSCs on a Chit/Glu/HA scaffold consisting of a hybrid polysaccharide matrix composed of chitosan and β-1,3-glucan and bioceramics were found to produce more Col I and exhibit higher OCN expression and mineralization intensity than BMSCs after long-term (28 days) osteogenesis induction [114]. This is unexpected given that BMSCs typically exhibit superior osteogenesis to ADSCs. Previous short-term (6 days) results demonstrated that BMSCs on the surface of this material had better osteogenic performance [23]. The disparate results may be attributable to the various ADSC sources and experimental periods. Undoubtedly, it is essential to examine the long-term effects of materials on ADSCs.

In light of the potential effects of surface topography on stem cell differentiation, it is anticipated that bioceramics scaffolds with a unique surface topography will be combined with ADSCs for BTE. However, as a result of the brittleness and poor machinability of bioceramic scaffolds, the preparation of adjustable surface topography remains a challenging problem that is currently understudied. Recently, the hexagon-like column array surface and natural island morphology were successfully fabricated on the surface of HAp scaffolds using a hydrothermal approach as well as a casting technique, respectively. These surfaces enhanced ADSCs’ adhesion and osteogenic differentiation significantly [57]. In particular, HAp scaffolds with isolated island surfaces significantly promoted the expression of ADSCs’ cytoskeletal organization, focal adhesion molecule and key osteogenic markers [57]. Correspondingly, the expression of BMP-2 was increased significantly, suggesting that BMP/Smad signaling pathway is activated and contributes to the osteogenic differentiation of ADSCs [57].

In addition, when selecting a combination of ceramics and polymers, compatibility and interaction must be carefully considered. Due to the lack of interaction between the materials, the mechanical properties of the osteogenic differentiation platform of ADSCs prepared with BGs and PLA decrease with decreasing BG content. In addition, the surface of the scaffold demonstrated strong hydrophobicity [112]. On the contrary, the combination of poly(D,L)-lactic acid (PDLLA) and β-TCP yielded optimal results. Due to the strong interaction between β-TCP and PDLLA, the composite scaffold demonstrated suitable porosity and better mechanical properties. In addition, it demonstrated adaptability to ADSCs seeding, as reflected by the electron microscope results after 5 days of culture. Furthermore, the scaffold significantly increased the expression of osteogenic genes of ADSCs, including ALP, BMP-2, Col I and RUNX2 [115].

### Polymeric biomaterials

#### Synthetic polymers

It has been reported that PCL has a regulating effect on stem cell behavior, particularly directional differentiation [116, 117]. For instance, PCL scaffold prepared by 3D printing technology was shown to significantly promote the proliferation, ALP expression and calcium deposition of ADSCs [62]. Moreover, it was discovered that the molecular weight of PCL significantly affected scaffold-cell interaction by altering the hydrophilicity and mechanical properties of scaffold surfaces. Similar to other lineage cells, ADSCs proliferate and differentiate into osteoblasts more effectively on scaffold surfaces with increased surface hydrophilicity and enhanced mechanical properties. Thus, a low molecular weight PCL scaffold offers significant promise as a kind of BTE material when combined with ADSCs [62]. However, it cannot be overlooked that PCL materials devoid of binding motifs do not promote cell adhesion and spreading [118, 119]. In addition, pure PCL scaffolds are comparatively soft. In light of the influence of mechanical compatibility on the phenotypic transformation of stem cells, biomechanical properties that are subpar may have negative effects on cell differentiation [120, 121]. In order to improve cell behavior on PCL-based scaffolds, published reports have attempted to manufacture composite scaffolds with superior biological properties by incorporating other polymers and/or ceramics into the PCL matrix. By combining PCL and protein, the hydrophilicity and surface biological reactivity of PCL materials can be enhanced, which is advantageous for their application in conjunction with ADSCs. In this regard, it has been reported that a PCL/Col composite scaffold promotes ADSC adhesion, proliferation and osteogenic differentiation. This is largely due to the addition of ECM protein Col, which provides polypeptide binding motifs. In this foundation, magnetic nanoparticles (MNPs), which were proved to promote osteogenic differentiation and inhibit adipogenic differentiation of ADSCs, were introduced into PCL/Col scaffold. The osteogenic differentiation of ADSCs was more significantly promoted on the PCL/Col/MNPs scaffold with great hydrophilicity and surface roughness, even in osteogenic cues-free media conditions [24]. In addition, it has been demonstrated that the natural protein zein increases the hydrophilicity of PCL fiber and produces a surface with a high cation distribution. Therefore, ADSCs adhered, grew and spread more actively on the surface of the PCL/zein fiber scaffold [122]. Polysaccharide improved PCL’s biological reactivity significantly [123, 124]. Not all polysaccharides could, however, promote the osteogenic differentiation of ADSCs. Thus, selecting the appropriate polysaccharide components was highly crucial [123]. PCL/polysaccharide composite scaffolds prepared by adding 10% w/w marine sulfated polysaccharide (ulvan alone or in blends with -carrageenan or/and chondroitin sulfate) possess a porous structure and high hydrophilicity. Unfortunately, neither κ-carrageenan nor chondroitin sulfate significantly enhanced the osteogenic response of ADSCs. In contrast, the PCL/ulvan scaffold with the highest ulvan content promoted ADSC proliferation, adhesion, diffusion and osteogenesis significantly [123]. At the same time, PCL/ulvan scaffolds with different pore structures exhibited equivalent osteogenic induction ability, further demonstrating the predominance of polysaccharide components in ADSCs’ cell behavior [123]. In an effort to provide cells with a greater capacity for osteogenic induction, ceramic materials were incorporated into PCL materials.

It was determined that PLGA was an appropriate ADSCs carrier. Sixteen weeks after the PLGA containing ADSCs was implanted into a rat calvarial defect model, new bone formation was stimulated [125]. However, the PLGA-only surface of the scaffold did not have sufficient biological induction effect. For PLGA to be combined with stem cells for BTE, its surface must be modified with additional materials. In this context, nanoparticles have been frequently used to modify the surface of PLGA scaffolds. Nanoparticles are a popular choice for surface modification of PLGA scaffolds. PLGA modified by GNPs and IONPs such as γ-Fe_2_O_3_ and α-Fe_2_O_3_ has been proved to promote the osteogenic behavior of ADSCs [70, 126, 127]. Interestingly, despite similar surface properties, PLGA-based scaffold modified by γ-Fe_2_O_3_ had a stronger ability to promote ADSCs osteogenic differentiation than those modified by GNPs (Figure 4B) [126]. This is primarily attributable to the magnetic effects of γ-Fe_2_O_3_, as indicated by the increased expression of exogenous magnetoreceptor (iron–sulfur cluster assembly protein 1), which is highly correlated with the expression of osteogenic genes [126]. This magnetic effect of IONPs has been proven to induce stem cell differentiation [128]. In addition to the beneficial effects of the inherent biological activity of IONPs on the behavior of ADSCs brought by magnetic effect, the incorporation of nanoparticles into other biomaterials improves the mechanical strength, surface wettability and roughness of the biomaterials, thereby creating a more favorable biophysical environment for the osteogenic differentiation of ADSCs. PCL-based materials modified by α-Fe_2_O_3_ had weak magnetism; however, the improvement of physical and chemical properties doubled the mineralization ability of ADSCs [127]. Moreover, the application of IONPs had special effects on ADSCs adhesion. Nanoparticles significantly promoted the initial adhesion and cell-fiber entanglement of ADSCs by providing more adhesion sites [127]. It was found that γ-Fe_2_O_3_ promoted the expression of integrin β1 of ADSCs on the surface of PLGA-based scaffold, which is essential for cell adhesion and subsequent osteogenic differentiation [70].

#### Nature polymers

As stated previously, collagen scaffolds are exceptionally biocompatible and biomimetic [129]. The collagen-based microcarrier was shown to be a suitable delivery tool for ADSCs in terms of cell adhesion, survival, proliferation and migration within scaffold pores [130]. Most importantly, the scaffold improved ALP expression and ECM mineralization of ADSCs, indicating a positive effect of the scaffold on osteogenic differentiation of ADSCs [130]. However, because of the low density of collagen material, it lacks the mechanical strength required for BTE. Thus, utilizing biaxial plastic compression, a Col scaffold with a compact structure was successfully created [131]. The results indicated that collagen fibers in the scaffold were densely and systematically arranged, and the hardness in the direction of fiber arrangement was more than 100 times than in the transverse direction, indicating that the mechanical strength was anisotropic. This structure and anisotropy of mechanical properties led to ADSCs’ favorable growth and spindle-shaped extension along the direction of fiber arrangement [131]. Although the mechanical strength of the scaffold was able to fully support its application in tendon and ligament repair, it is unclear whether a compact collagen material suitable for orthopedic implants can be prepared. Gelatin, which is derived from collagen, is a popular choice for BTE combined with seed cells due to its low immunogenicity and biological reaction activity [132]. ADSCs were seeded onto an epigallocatechin gallate-modified gelatin sponge scaffold, incubated for 24 h and then implanted into a rat congenial cleft-jaw model [133]. The combination of ADSCs and gelatin sponge stimulated bone formation at the implant site after 4 weeks. Experiments *in vitro* demonstrated that the gelatin sponge’s hydrophilicity, negative charge surface and calcium phosphate precipitation created a conducive microenvironment for ADSC-mediated bone regeneration *in vivo* [133].

As one of the primary glycosaminoglycans in tissues, HA is the most extensively studied polymer in the development of hydrogel for the 3D culture of encapsulated stem cells. Hyaluronidase, which is secreted by numerous cells, including stem cells, can induce the degradation of HA and is regarded as a crucial environmental cue for stem cell activity [134]. Nonetheless, pure HA hydrogels lack the necessary mechanical properties to support the differentiation of stem cells into bone tissue structure [135]. Thus, using HA-based hydrogels to support ADSCs activity and adding fragmented PCL nanofiber to HA to improve its mechanical properties, a type of hybrid scaffold material was developed to meet the needs of BTE [135]. ADSCs encapsulated in the hybrid scaffold material containing 20% nanofiber exhibited good osteogenic differentiation, as indicated by the expression of osteogenic markers Col I, ALP and RUNX2, and by the mineralization of ECM [135]. In addition to the above, alginate beads have also been demonstrated to function as suitable structures for the development of multiscale 3D culture systems for stem cells [136]. It has been reported that alginate-based hydrogel microspheres with gelatin and functionalized mesoporous silica nanoparticles provide an excellent microenvironment for the growth, osteogenic differentiation and ECM mineralization of ADSCs [137]. Moreover, ADSCs spheroids have been successfully formed on cellulose hydrogel film in recent years [138]. This self-assembled cell mass has a similar morphology and function to natural tissues, making it a promising model for cell carriers. The spheres of ADSCs remained stable for 7 days and demonstrated enhanced osteogenic differentiation capacity. However, the regulation of sphere size, which was positively correlated with cell seeding density and culture time, and the more detailed bone regeneration effect warrant additional research [138].

### Decellularized extracellular matrix

Although a variety of tissue engineering scaffolds, such as metallic, ceramic and polymeric biomaterials, have been widely used, these materials struggle to overcome immunogenicity, mimic the microenvironment of natural bone tissue and exhibit mechanical properties similar to those of individual organs or tissues [19]. The emergence of dECM scaffold offers a novel approach to overcoming these obstacles. Due to the fact that it simulates the natural cellular microenvironment with its natural 3D structure, various trace elements and bioactive components, it serves as a substitute scaffold for bone regeneration [139]. In particular, the physical and chemical signals and biological characteristics of dECM can still be retained after decellularization, providing a substrate for mechanical support and a biological 3D carrier for subsequent cell inoculation [140]. As sources of inspiration for the repair of bone defects, acellular scaffold and stem cell technology cannot be ignored in the field of tissue engineering at this time.

Depending on the source of the ECM, dECM scaffolds can be classified into two categories: O/T-derived dECM scaffolds and cell-derived dECM scaffolds [19]. O/T-derived dECM scaffolds preserve tissue-specific memory factors and complex ECM structures, which can stimulate tissue-specific differentiation and regulate cell adhesion and proliferation [141]. In 2019, a study encapsulated decellularized adipose tissue and decellularized trabecular bone with hADSCs in hydrogels to compare the osteogenic capability of hADSCs after decellularization and recellularization in growth or osteogenic medium [142]. Decellularized trabecular bone could more effectively promote the osteogenic differentiation of ADSCs when interacting with soluble factors exerting in OM [142]. In a separate study, artificial scaffolds coated with decellularized and demineralized ECM were used to repair mandibular defects in beagle dogs [143]. Eight weeks after the intervention, the ADSC-containing group exhibited more diffuse osteoblast tissue. It is worth mentioning that, unlike the traditional method of directly seeding cells on the surface, the cell suspension of ADSCs was injected into the pores of the scaffold [143]. This method not only significantly increased the cell seeding density but also enhanced the paracrine response, thereby increasing the paracrine factors that stimulated cell proliferation and bone regeneration [143]. As the most prevalent bioink for 3D printing, alginate could provide exceptional printability due to its rapid gelation, but its weakness was its lack of bioactive components that affect the biological activities of cells laden in scaffolds [144]. To overcome the limitations, dECM derived from porcine bone tissue (Ma-dECM) was added to an alginate-based cell-rich composite [145]. Multiple experimental results demonstrated that the composite significantly stimulated osteogenesis. Nevertheless, a higher concentration of Ma-dECM was capable of inducing a variety of exceptional cellular activities, but it significantly reduced *in situ* cell viability during the printing process due to its high viscosity [145]. Consequently, selecting the proper concentration of dECM to exert the biological activity of scaffolds is also a crucial step [145].

Compared to O/T-derived dECM scaffolds, cell-derived dECM scaffolds are easier to produce [19]. It is available from certain progenitor cells and stem cells and is able to reduce the risk of pathogen transfer caused by allogeneic ECMs and eliminate the adverse host immune responses induced by xenogenic ECMs [146]. The cell-laid matrix refers to the dECM coating on the scaffold. It is secreted by the cells that are initially seeded on the scaffold and undergoes decellularization and recellularization [147]. Using this technique, researchers obtained the acellular functionalized PCL scaffold (aoPCL), and inoculated hADSCs on its surface. They discovered that the composite could stimulate hADSCs to produce calcium and that all genes relevant to osteogenesis were significantly overexpressed on aoPCL compared to unmodified PCL [148]. Although the current study demonstrated a positive effect of the aoPCL scaffold on the differentiation capacity of hADSCs, only an *in vitro* evaluation was conducted. Therefore, future *in vivo* studies are required to determine the clinical applicability of the scaffolds and their effect on the tissue regeneration potential of hADSCs.

## **A**DSC-loaded scaffold materials combined with **bioactive molecules** for bone regeneration

As demonstrated in the preceding section, various biomaterials and their complexes play a crucial role in all phases of bone defect repair. However, abundant BMs, such as BMP-2, PDGF-BB, ADSCs-exo, inorganic ions and small molecule drugs, have also been used to enhance bone repair. When these molecules are applied to ADSCs alone or in conjunction with biomaterials at appropriate release rates within the concentration range, the osteogenic differentiation of ADSCs is significantly enhanced, thereby promoting bone tissue formation (Table 3).

**Table 3.** Osteogenesis of ADSC-loaded materials combined with **bioactive molecules**

BMs		Carrier	Cell	Medium	Model	Osteogenesis-related change	Ref.
BMP-2	BMP-2	BOPSC	mADSCs	basal medium	C57BL/6 male mice	Migration (+); Proliferation (+); ALP activity (+); Expression of RUNX2, OPN, Col I and OCN (+), BSP (−)	[28]
	BMP-2	PLGA/PCL	rADSCs	OM	NA	Adhesion (+); Proliferation (+); Calcium deposition level (+); Expression of ALP, Col I, MSX2, OCN (+)	[29]
	BMP-2	Gelatin-based μRB hydrogel	mADSCs	OM	FVB mice calvarial defect	Proliferation (+); Collagen deposition (+); Bone mineral formation *in vivo* (+)	[73]
	rhBMP-2	Ti-pGMA	hADSCs	OM	NA	ALP activity (+); Calcium deposition level (+); Expression of OCN, RUNX2, Col I (+)	[151]
PDGF	PDGF-BB	HC-DCB-PCL	ADSCs	OM	Murine calvarial defect	Mineralization (+); Calcium deposition level (+); Expression of RUNX2, OCN, OPN (+); Bone mineral formation *in vivo* (+)	[32]
FGF	FGF-2+A2-P	PPX	hADSCs	OM	Rat calvarial defect	Expression of OCN (+); Bone tissue deposition *in vivo* (+)	[30]
	FGF+HGF	HAp/β-TCP	ADSCs-E	OM	Balb/c nude mice	Expression of OCN (+); New bone regeneration *in vivo*	[9]
Exosome	ADSCs-exo	Ti	hADSCs	OM	NA	Adhesion (+); Migration (+); Proliferation (+); Expression of RUNX2 (+)	[160]
	ADSCs-exo	PLA	hADSCs	MSCGM	NA	Expression of OPN, Col I, OCN and ON (+)	[162]
Inorganic ions	Li^+^	nHAp	hADSCs	OM	NA	Calcium deposition level (+); Expression of BMP-2, OPN and OCN (+), GSK3β (−)	[164]
	Mn^2+^+BG	TiO_2_	ADSCs	OM	NA	Adhesion and spreading (+); ALP activity (+); Calcium deposition level (+); Expression of OCN (+)	[111]
	Mg^2+^+Na^+^	MgSnZnNa	mADSCs	OM	NA	Expression of OCN and OSX (+)	[17]
Small molecule drugs	AT	PLGA-HAp	ADSCs	OM	NA	Mineralization (+); Calcium deposition level (+)	[169]
	Simvastatin	0.5%nSi nanocomposite hydrogels	hADSCs	OM	NA	Migration (+); ALP activity (+); Expression of RUNX2 (+)	[170]
	RSV	PVA-SF	hADSCs	OM	NA	Calcium deposition level (+); Expression of RUNX2, ALP, OPN, and OCN (+)	[176]
	ALN	Chitosan	hADSCs	OM	NA	ALP activity (+); Calcium deposition level (+); Expression of OPN (+)	[10]
Other bioactive substances	hUCS	Col/α-TCP	hADSCs	OM	Mastoid obliterated rat	Proliferation (+); Calcium deposition level (+); Expression of RUNX2, ALP, OPN, and OCN (+); New bone regeneration *in vivo*	[180]
	hPE	Gel/HA	hADSCs	NA	NA	Proliferation (+); Expression of ALP, RUNX2, BMP-2, OPN, and OCN (+)	[182]

‘(+)’ represents expression is up-regulated or promoted; ‘(−)’ represents expression is down-regulated or inhibited; NA, not applicable.

### Growth factors

As a type of signaling molecules, GFs regulate not only the physiological processes of cell proliferation, differentiation and migration but also the formation of vital tissues such as bone and blood vessels [149]. BMP is a member of the TGF-β superfamily in BTE, which comprised primarily of BMP-2, BMP-7 and BMP-9 [150]. In particular, the strong osteoinductive properties of BMP-2 have been extensively studied [28]. To release BMP sustainably for effective bone regeneration based on ADSCs, numerous delivery systems composed of various materials have been studied [28, 151]. It is common knowledge that phospholipid, the principal component of the cell membrane, is an amphoteric molecule with a hydrophilic end and a hydrophobic end [152]. Inspired by this, researchers chose to cover the hydrophilic segment of BMP-2 with soybean lecithin, and the exposed hydrophobic segment was combined with polyester material to create the bioactive osteo-polyester scaffold (BOPSC), which exhibited a high retention efficiency (95.35%) and continuous release of BMP-2, thereby producing an excellent osteogenic microenvironment [28]. When the scaffold was implanted into the back of mouses, researchers discovered that the chemotactic ability of mouse ADSCs (mADSCs) to the bone defect site was significantly enhanced, as were their proliferation and migration capacities [28]. In addition, in a separate report, glycidyl methacrylate (GMA) was deposited on the surface of titanium and then chemically coupled with rhBMP-2 via a ring-opening reaction [151]. The addition of rhBMP-2 enhanced the hydrophilicity of the implant, which was essential for a more efficient interaction between hADSCs and the Ti surface [153], thereby significantly enhancing hADSCs adhesion, migration and proliferation. Moreover, the effective release of rhBMP-2 created an ideal microenvironment for promoting osteogenesis in hADSCs [151]. Different from the above fixatives, some researchers combined bovine serum albumin with BMP-2, thereby immobilizing BMP-2 on the fiber scaffold [29]. Similarly, sustained release of BMP-2 enhanced the osteogenic differentiation of rADSCs cultured on the scaffold. In addition, they created a 3D structure for the scaffold. Interestingly, the synergistic effect between BMP-2 and the physical-mechanical properties of the 3D scaffold further enhanced the cell–cell and cell–matrix interaction, and had a positive effect on the osteogenic differentiation of rADSCs [29]. It can be seen that the 3D structure and the scaffold containing GFs have great potential for osteogenesis of ADSCs. Similar to the previous study, another study in which BMP-2 was directly coated onto the surface of a scaffold with protein demonstrated that the fibrinogen coating physically absorbed the BMP-2 that was effectively released [154]. When applied to a mouse calvarial defect model, the released BMP-2 and ADSCs caused a strong deposition of collagen and significantly sped up bone regeneration [73].

ADSCs require a supraphysiological dose of BMP-2 to induce bone regeneration when the trauma exceeds critical-sized defects, but their clinical application is limited by concomitant side effects, such as ectopic bone formation, inflammation and bone resorption [155]. In a 2014 study that combined BMP-2 with a small amount of PDGF-BB acting on ADSCs, PDGF-BB was found to be promising in promoting the osteogenic differentiation of ADSCs [156]. A year later, it was discovered that PDGF could directly promote osteogenesis of ADSCs through PDGFR signaling and was more effective than BMP-2 at the same concentration [144]. PDGF-BB was immobilized on decellularized bone particle (DCB) scaffold, and the results showed that it promoted the osteogenic differentiation of ADSCs more consistently than scaffolds lacking PDGF-BB [32]. Interestingly, scaffolds containing PDGF-BB mineralized earlier than those without it. It was hypothesized that PDGF altered the kinetics of osteogenic differentiation because the osteogenic gene was upregulated earlier [32].

In addition to the two cytokines mentioned above, numerous studies have confirmed the importance of FGF-2 in bone development and maintenance [30]. FGF-2 exerts local effects in the bone matrix by binding to FGF receptors 1 and 2 to form dimers, and then initiating the Wnt signaling pathway via PI3K in osteoblasts and MSCs [157]. The multi-component PPX scaffold containing hADSCs and BMs (FGF-2 and A2-P) was used to study the calvarial defect in rats. Multiple visible bone tissue deposits within the cranial cavity were strong evidence of effective bone regeneration [30]. In addition, it was determined that the combination of FGF and HGF could enhance the differentiation capacity of hADSCs [9]. Compared to young adults, ADSCs in the elderly (ADSC-E) have a relatively low differentiation potential, slow proliferation, inadequate paracrine ability and age rapidly [158]. After numerous experiments, researchers chose FGF and HGF, two age-related osteogenic soluble factors, to bind to the scaffold as BMs [9]. ADSC-E that had been pretreated with FGF/HGF were inoculated onto HAp/β-TCP, and the scaffold was then transplanted onto the backs of null mice, where significant bone-like structure and new bone formation were observed. The observable evidence suggested that the combination of the two factors significantly improved the osteogenic capacity of ADSC-E [9]. The study provided a new strategy for treating bone diseases in elderly patients. Thus, the combination of FGF-2/HGF and biomaterials for bone regeneration in elderly patients is likely to be a potential research direction for future studies.

### ADSCs-exo

As stated previously, ADSCs-exo, a new bone-inducing factor, has received considerable attention in recent years due to its ability to induce osteogenic differentiation in MSCs [159, 160]. In previous experiments, BMSCs were used as seed cells to test the osteogenic effects of ADSCs-exo [103]. However, some researchers discovered that ADSCs can absorb numerous ADSCs-exo within 6 h, whereas BMSCs require 48 h [161], possibly because ADSCs-exo is more readily absorbed by homologous cells. This informs us that the combined use of ADSCs-exo and ADSCs in BTE can reduce the loss of exosomes due to the long absorption time, thereby promoting bone regeneration more effectively. Thus, immobilized ADSCs-exo significantly promoted the proliferation and extension of hADSCs adhered to a Ti scaffold [160]. Excellent cell compatibility is a prerequisite for osteogenesis. Another study confirmed ADSCs-exo’s pro-osteogenic effect on ADSCs [162]. The experiments revealed that exosomes are readily captured on the surface of scaffolds, and their existence improved the gene expression of osteogenic marker [162]. In summary, the attachment of exosomes to ADSC-loaded scaffold materials, which provides a favorable environment for exosomes to exert their osteogenic function, is a popular and promising treatment method for bone defects in current and even future research.

### Inorganic ions

Inorganic ions exert crucial regulatory effects during the formation of bone. For instance, the release of lithium ions (Li^+^), silicon ions (Si^4+^), manganese ions (Mn^2+^) and iron ions (Fe^3+^) on the bone repair scaffold upregulates the osteogenic capability of cells and promotes bone tissue regeneration [70, 111, 163, 164]. Li^+^ is primarily used in medicine for the short-term or long-term treatment of depression and mania [164]. Interestingly, the bone and mineral density were found to increase in a patient population treated with lithium [163]. Thus, Li^+^ has become an additional strategy for bone regeneration treatment. A study published in 2017 demonstrated that ADSCs cultured on a nanohydroxyapatite (nHAp) scaffold containing Li^+^ had increased viability and proliferation [165]. Later, the same group of researchers examined the adhesion and osteogenic differentiation capabilities of ADSCs. They discovered that Li^+^ increased the expression of OPN, which sheds a hopeful light on Li^+^ and nHAp construct’s ability to promote cell adhesion, which is essential for the initiation of the regeneration process [164]. Furthermore, the decreased expression of glycogen synthase kinase 3β (GSK3β) and the increased expression of β-catenin confirmed the conclusion of previous researchers that Li^+^ promoted bone formation by activating the Wnt/β-catenin signaling pathway and inhibiting GSK3β [166]. As can be seen, Li^+^ is an effective method for bone regeneration, and its future potential is promising. Manganese (Mn) is an essential cofactor for numerous enzymes and participates in the synthesis of glycosyltransferase and chondroitin sulfate during the formation of the bone matrix [167]. According to a study in 2021, BG containing Mn^2+^ promoted the osteogenic lineage differentiation of equine ADSCs [168]. Some researchers modified titanium with manganese-containing BG and investigated the adhesion, proliferation capability and osteogenic effects of the composite scaffold on ADSCs [111]. Si^4+^ released by BG promoted the differentiation of ADSCs and played an early role in the formation of bone matrix. In addition, Mn^2+^ is indispensable for enhancing cell growth and bone mineralization [111]. Recently, a novel Mg alloy containing the essential element sodium was developed and demonstrated to be superior to conventional Mg alloys in terms of bone regeneration [17]. Experiments *in vitro* confirmed that a certain concentration of Mg^2+^ and Na^+^ significantly increased the expression of OCN and osterix (OSX) in ADSCs [17]. This function was accomplished in the standard growth medium. However, the direct impact of ion co-release on ADSCs has not been investigated. The effect of this material on ADSCs and the possibility of combined *in vivo* application require further investigation [17]. To summarize, the combination provides a material with excellent osteogenic potential.

### Small molecule drugs

At present, many drugs are used as substitutes of GFs for stem cell recruitment, statins, such as atorvastatin (AT), simvastatin and rosuvastatin (RSV), and bisphosphonates such as alendronate (ALN) are of great interest in this field [169, 170]. Small molecule drugs are well-known for their ability to reduce cholesterol levels. People have been deeply concerned about the use of statins for new bone formation rather than lipid-lowering therapy in recent years [171]. Correspondingly, AT has been shown to stimulate osteogenic differentiation of BMSCs, promote bone formation and inhibit osteoclast-mediated bone resorption [172]. Particularly, a study on an AT-loaded scaffold material using ADSCs as seed cells revealed that a small amount of AT could induce a substantial amount of calcium deposition, demonstrating its high potential for osteogenic differentiation [169]. As a statin with the same hydrophobicity, simvastatin promotes the osteogenic differentiation of stem cells by increasing the expression levels of ALP and BMP, and has a promising future application in bone regeneration [173]. In an intriguing study, simvastatin was loaded into a 0.5% silicate-based nanocomposite hydrogel and its osteogenic potential for hADSCs was monitored *in vitro* [170]. The released simvastatin not only maintained the chemotaxis of hADSCs, causing their migration to the regions with higher simvastatin concentrations, but it also stimulated the osteogenic differentiation of hADSCs [170]. However, compared to hydrophobic drugs, hydrophilic RSV had fewer adverse effects and a longer terminal half-life [174]. As reported, RSV at a suitable dose had potential effects to affect osteogenesis and bone regeneration [175]. In the experiment, it was also determined that hADSCs cultured on RSV-loaded nanofibers had a greater capacity for cell proliferation, and that RSV effectively induced the osteogenic differentiation of hADSCs by stimulating the expression of RUNX2 [176]. In addition to statins, another small molecule drug is bisphosphonates, with ALN serving as its representative drug. As a third-generation bisphosphonate, ALN has an anabolic effect by promoting osteoclast apoptosis, osteoblastogenesis and osteoblast maturation [177, 178]. Currently, ALN is being utilized as a therapeutic agent in a number of studies evaluating the osteogenic differentiation effect of ADSCs. As evidenced from earlier studies, ALN-loaded chitosan nanoparticle delivery systems have been manufactured [10]. Compared to bare nanoparticles, the significantly increased ALP and calcium contents demonstrated that ALN had a strong ability to promote the osteogenic differentiation of hADSCs, thereby making it a suitable compartment for BTE scaffolds [10].

### Other bioactive substances

Human-derived materials used as GF supplements have demonstrated therapeutic effects in various eye diseases, but little research has been conducted on bone regeneration [179]. To enhance the osteoinductive and osteoconductive properties in ADSC-loaded scaffold materials, hUCS (which contains various GFs, including VEGF, TGF-β, bFGF) was added to Col/α-TCP scaffolds loaded-with hADSCs [178, 180]. The results demonstrated that hADSCs exhibited significantly enhanced osteogenic properties *in vitro* and induced osteogenic activity in a mastoid-deficient rat model [180]. Surprisingly, hPE and hUCS were found to share similar properties. Besides numerous GFs, hPE also contains poly deoxynucleotide, which is a potential stimulus to osteogenesis and angiogenesis [181]. On this basis, the biocomposite composed of hPE, hADSCs and gelatin/HA was manufactured. The *in vitro* results demonstrated that hPE could increase the proliferation of hADSCs, and the increase in ALP activity indicated that hPE also had the potential to promote the osteogenesis of hADSCs [182]. This is primarily due to the synergistic effect of the various bioactive factors in hPE.

## Application of ADSC-loaded scaffold materials for bone regeneration: clinical studies

### Clinical case reports using ADSC-loaded scaffold materials in bone reconstruction

Although cell therapy has shown the potential to promote bone regeneration in preclinical studies, further clinical researches are needed to comprehensively evaluate the cell source, operation method, mode of application and cost-effectiveness before cell therapy becomes a routine clinical procedure [183]. Lendeckel *et al*. published the first report of craniofacial repair using ADSCs in 2004. A 7-year-old girl suffered from widespread calvarial defects due to chronic infection after severe head injury. Autologous ADSCs were applied to the calvarial defects and were kept in place by using autologous fibrin glue. CT-scans showed new bone formation 3 months after the reconstruction. The successful results demonstrated the beneficial effect of autologous ADSCs on craniofacial repair for the first time (Table 4) [184]. Subsequently, increasing studies have shown that ADSCs loaded on different types of composite biomaterials had a significant enhancement on bone regeneration [185–187]. Some academics combined unmodified autologous adipose-derived regenerative cells (UA-ADRCs) and fraction 2 of plasma rich in growth factors (PRGF-2), loading them on an osteoinductive scaffold (OIS) for the first time. It was demonstrated that the combination of UA-ADRC/PRGF-2/OIS resulted in better and faster osseointegration than the combination of PRGF-2 and OIS alone (PRGF-2/OIS) in maxillary sinus augmentation and lateral alveolar ridge augmentation [185]. Autologous ADSCs were seeded in β-TCP granules, with or without resorbable mesh in bilaminate construction, and the ADSC-loaded scaffolds were implanted in four patients who had large calvarial defects of different etiologies. The good news was that there were no signs of clinical complications in any patients at 3 months postoperatively [186]. In another research, patients with large craniofacial osseous defects were included. All 23 patients were reconstructed with ADSCs, resorbable scaffolds (beta-TCP or bioactive glass) and GF (rhBMP-2). Preliminary results and clinical observations were extremely encouraging, since only one case of infection and two of inadequate bone formation [187]. Moreover, hADSCs were also loaded on the HAp-Col hybrid scaffold named Col I/Pro Osteon 200. During a 3-year follow-up, a comprehensive evaluation was performed among 50 patients who underwent zygomatic augmentation and bimaxillary osteotomy. It was demonstrated that histological results of biopsy specimens showed prominent ossification, suggesting the excellent osteoinductivity of this kind of ADSC-loaded scaffold materials [188].

**Table 4.** Published clinical case reports using ADSCs in bone tissue engineering

Condition	Cell	Scaffolds	Follow-up	Result	Ref.
Calvarial defects	Autologous ADSCs	Milled bone from iliac crest with autologous Fibrin glue	3 months	New bone formation and complete calvarial continuity after reconstruction	[184]
Partly failing maxillary dentition	UA-ADRCs	OISs	6,34 weeks	Excellent bone healing	[185]
Calvarial defects	Autologous ADSCs	β-TCP granules	3,12 months	No signs of complication in all patients; The calvarium was firm	[186]
Craniofacial osseous defects	Autologous ADSCs	Resorbable scaffolds (β-TCP or BG)	NA	23 cases were successfully implanted into humans to reconstruct their jaws, but one infection and two cases with inadequate bone formation	[187]
Malar augmentation procedures	Autologous ADSCs	HAp-Col hybrid scaffold	1,24,36 months	The material has been resorbed and replaced by cortical bone	[188]
Maxillary defects	GMP-level autologous ADSCs	β-TCP particles	8 months	The flap had developed mature bone structures and vasculature	[191]
Cranio-maxillofacial defects	GMP-level autologous ADSCs	β-TCP or BG	12–52 months	10 cases achieved successful integration of the construct to the surrounding skeleton; Two cases were used sustained bone resorption; One case failed outright at 1 year	[192]
Anterior mandibular amelo-blastoma resection defect	GMP-level autologous ADSCs	β-TCP granules	10 months	Successful rehabilitation with dental implants	[193]
Calvarial defects	Autologous ADSCs	β-TCP granules	6 years	Three patients developed poor ossification and another was reoperated for meningioma	[197]

NA, not applicable.

Despite ADSCs are being proven to be suitable candidates for clinical bone tissue reconstruction, they cannot be directly applied to clinical populations, based on the current regulatory issues [189]. There is clearly a need to standardize the method of cell preparation for the use in regulated clinical setting, in order to prevent cell from being polluted during preparation. The national regulatory agencies requiring good manufacturing practice (GMP) to be fulfilled during cell therapy [190]. Mesimäki *et al*. isolated and amplified ADSCs according to the GMP standard for the first time. The study described a novel method to reconstruct major maxillary defects using GMP-grade human autologous ADSCs combined with rhBMP-2 and β-TCP particles. After 8 months of follow-up, the flap had developed mature bone structures and vasculature. The successful outcome of this clinical case paves the way for extensive clinical studies of GMP-grade ADSCs for the treatment of craniofacial bone defects [191]. Similar promising results were observed in another study; GMP-level autologous ADSCs were used in the treatment of cranio-maxillofacial defects as well. A review recorded 13 consecutive cases of cranio-maxillofacial defects at frontal sinus (three cases), cranial bone (five cases), mandible (three cases) and nasal septum (two cases). The defects were reconstructed with either BG or β-TCP scaffolds seeded with ADSCs. Successful osseointegration was noted in 10 of the 13 cases during the follow-up time ranging from 12 to 52 months [192]. The same team used a tissue-engineered construct consisting of β-TCP granules, BMP-2 and GMP-level ADSCs to replace a 10-cm anterior mandibular amelo-blastoma resection defect. Successful rehabilitation with dental implants was observed 10 months after reconstruction [193]. The same biomaterials and GMP-level ADSCs, with progressively increasing usage of computer-aided manufacturing technology, were used in another three patients. The ideal reconstruction of large mandibular defects, together with rapid prototyped reconstruction hardware supporting placement of dental implants, was observed after 10 and 14 months following reconstruction as well [194].

In general, ADSCs need to be expanded *in vitro* for 2–3 weeks before implantation to achieve appropriate cell yield, and the whole *in vitro* expansion procedure and relative reagents used must comply with GMP standards [189, 195]. Nevertheless, *in vitro* ADSCs amplification not only requires multiple surgical interventions [195] but also may attribute to genomic instability and further structural or digital chromosome aberrations [195]. Moreover, such cumbersome regulatory procedures have also become a major obstacle on the road to clinical translation of stem cell therapy. Therefore, a growing number of researchers have begun to explore alternative cell therapies instead of ADSCs amplification *in vitro*.

In the light of the above researches, it is not difficult to conclude that ADSC-loaded scaffold materials exhibit great potential for osteo-inductive efficacy. Unlike preclinical studies, however, clinical studies should focus more on the safety of ADSCs in patients with bone-related diseases, in addition to the *in vivo* bone regeneration potential [195]. Some patients who enter clinical research will inevitably have the outcome of infection and tumor recurrence due to complications such as diabetes and tumor [196]. Furthermore, the success of ADSC-loaded materials in reconstructive surgery has so far been limited to cases with a short follow-up period [191, 192, 194]. In a long-term follow-up report, five patients receiving cranioplasty used ADSCs, β-TCP granules and supporting meshes, similarly to the biomaterials mentioned above. The bone mineral density of the graft tended to increase <1 year after surgery. Nonetheless, the 6-year follow-up results of the five cases were not satisfied. Three patients developed poor ossification, and another patient was reoperated for meningioma [197]. This makes it difficult to draw reliable conclusions about the effectiveness of ADSCs in BTE and raises concerns about the safety and efficacy of ADSCs in clinical applications [197].

### Clinical trials using ADSC-loaded scaffold materials in bone reconstruction

The success of the clinical case reports has encouraged researchers to carry out further clinical trials on the implantation of biomaterials loaded with adipose stem cells. As of October 2022, we have retrieved the database on www.clinicaltrials.gov using the key search terms: ‘bone defect’ and ‘adipose cell’, with a total of three related clinical trials. These include one unknown status, one completed and one recruiting, with all three studies in Phase I/II (Table 5).

**Table 5.** Research on clinicaltrials.gov with the keywords ‘bone defect’ and ‘adipose cell’, resulting in three studies

Reference	Condition	Phase	Country	Status	Title
NCT02307435	Non-union fracture; Metaphyseal fibrous defect	Phase Ι	Indonesia	Unknown	Allogenic mesenchymal stem cell for bone defect or non union fracture (AMSC)
NCT02153268	Bone void in the maxillofacial area	Phase Ι/0	Israel	Completed	Filling bone defects/voids with autologous BonoFill for maxillofacial bone regeneration
NCT02842619	Bone augmentation; Bone grafting after removal of cysts from jaws	Phase Ι/0	Israel	Recruiting	Filling bone defects/voids with autologous BonoFill-II for maxillofacial bone regeneration

The earliest clinical trial (NCT02307435) started from May 2014 and lasted for up to 24 months. Researchers compared the effectivity of MSCs’ implantation from bone marrow, umbilical cord and adipose applied to non-union fracture and long bone defect. Surgical intervention was implemented for inserting the mixture of MSC and HA-CaSO_4_ into the defect area. Then, patient underwent clinical and radiological examination in the 3rd, 6th and 22nd month after implantation. Theoretically, ADSCs, as allogeneic stem cells, play a similar role to bone marrow-derived stem cells. Furthermore, ADSCs tend to exhibit greater advantages in clinic because they are more accessible and less traumatic to patients. Unfortunately, the result of this clinical trial has not been published. But even if the results are published, their significance is somewhat diminished by the fact that only one person was recruited for each MSCs (fat, bone marrow and umbilical cord) in the study. From June 2014 to January 2017, another clinical trial (NCT02153268) recruited 11 participants to evaluate the safety and the efficacy of BonoFill, an autologous human adipose tissue-derived cell-based product combined with OraGraft^®^ mineral particles. It was placed in the maxillofacial area for reconstructing the bone void with ∼6-month follow-up after implantation. Promising effects were shown. There was neither chronic bone infection nor significant changes in complete blood count and in general health, and the bone regeneration in the operated site was significantly accelerated. In July 2016, a similar clinical trial (NCT02842619) was in development based on a larger sample size study using the same artificial bone substitute called BonoFill. The recruitment of the trial is still ongoing. The participation of more patients will speed up the rate at which trials can be conducted, which is essential to advance the clinical application of biomaterials loaded with adipose stem cells.

### Challenges in clinical application of BTE loaded with ADSCs

In recent years, numerous studies on BTE loaded with ADSCs have demonstrated their potential for use in bone defect repair. Despite the abundance of relevant *in vitro* studies, experimental validation in animals is scarce, and clinical research is even more limited. The clinical transformation of BTE loaded with ADSCs still has much to discover and must overcome numerous obstacles.

Many factors need to be considered in the clinical application of ADSCs. The most basic problem is its security. At present, there are still great contradictions and disputes about the security of ADSC applications. Accordingly, numerous studies have shown that ADSCs may play a role in promoting cancer [198]. However, we know very little about the risks or adverse effects of ADSC-loaded BTE treatment due to the lack of animal and clinical experiments, particularly long-term experiments. Secondly, the current source selection of ADSCs is unclear. It should not be overlooked that the different sources of ADSCs significantly affect their proliferation, secretion, gene expression and osteogenic differentiation capacity, thereby significantly affecting the therapeutic effect of bone defects [199, 200]. More importantly, the isolation procedures, culture and amplification techniques, characterization methods, storage and transport of ADSCs are not standardized [201]. In addition, it is challenging to determine the optimal cell phenotype, number, and generation for different scaffolds and variable defect environments, which severely hinders the standardization process of clinical application of ADSCs [202]. Moreover, the establishment of regulatory guidelines, commercialization and cost control represents a final challenge in clinical application that cannot be ignored.

Regarding biomaterials, biological safety is also the primary requirement that must be met. Degradation of biomaterials will have a significant impact on their *in vivo* stability and regenerative capacity of tissues. Improper degradation rates and toxic degradation products will lead to harmful effects [203]. However, to achieve safe and effective tissue regeneration, it is difficult to precisely evaluate and control the biomaterials’ degradation *in vivo*. In addition, to ensure a good therapeutic effect, the mechanical properties and biological activity of biomaterials must correspond to the structural and repair requirements of various anatomical parts. Current research has difficulty achieving a perfect equilibrium between various performance requirements [204], and the successful clinical transformation of biomaterials cannot avoid the challenges of standard oversight, standard production and cost management [205].

With regard to BMs, security issues and cost effectiveness comprise the main challenges in their clinical application. Some studies have confirmed that GFs can cause bone resorption, inflammation, nerve damage and other adverse effects [206–209]. The tendency of GFs to undergo burst release brings potential off-target systemic effects *in vivo* [210]. In addition, BMs typically have a short active half-life, which significantly diminishes their therapeutic effects [202, 211]. Moreover, the delivery of large quantities of BMs may have minimal or even adverse effects. Thus, it is difficult to precisely regulate the release and delivery of BMs. Importantly, bone healing is a multi-stage, complex process during which the spatial and temporal characteristics of the release of various cytokines are distinct [212]. At present, the dynamic changes of various cytokines in the bone-healing process are not yet completely understood. Thus, it may be difficult to tailor the application of BMs to the temporal and spatial changes of bone healing, preventing them from exerting their maximum effect.

Combining ADSCs with bioactive materials and bioactive substances in BTE will face all the above challenges. Moreover, integrating the aforementioned three components is a complex and variable process. Repeated pre-clinical and clinical experiments are required to optimize and standardize the methods and processes of loading cells and BMs, *in vitro* culture, and characterization of scaffolds containing cells [6, 201]. It will be a lengthy process that requires step-by-step investigation. More importantly, the optimal combination of ADSCs with biomaterials and active substances is still undetermined. Numerous articles are devoted to the development of various biomaterials and attempts to combine them with ADSCs, but almost all of them remain at the level of simple attempts because they lack systematic verification, in-depth research and comparison. To realize the optimization of the treatment effect as quickly as possible, it is more efficient to compare different strategies to determine the optimal combination for more concentrated research.

## Conclusion and future perspectives

Numerous *in vitro*, *in vivo*, and preclinical and clinical studies have demonstrated the superiority of ADSCs in BTE. In addition to their exceptional osteogenic differentiation capacity, ADSCs also possess exceptional immunomodulatory and angiogenic abilities. The interaction between ADSCs and other cells at the implantation site, including immune cells and ECs, supports the formation of a regenerative environment. Taking into account the various osteogenic effects of ADSCs induced by various scaffold materials, selecting the appropriate scaffold material loaded with ADSCs will result in improved bone defect repair. In this regard, single-component biomaterials may not meet the requirements of BTE for biocompatibility, mechanical properties, degradation characteristics and biological activity. Conversely, composite biomaterials combining inorganic and organic components, particularly bionic materials with a unique surface morphology, can simulate the bone matrix environment more accurately and provide more abundant biological clues for the osteogenic differentiation of ADSCs. Moreover, the application of additional BMs will promote the bone repair effect of ADSC-loaded materials, which merits further investigation. It is, therefore, ideal to select a variety of BMs based on the release characteristics of cytokines during bone healing and to regulate their release in a sequential manner. Presently, a large number of articles are devoted to developing various biomaterials and BMs and attempting to combine them with ADSCs. Almost all these articles, however, remain at the level of simple attempts, lacking systematic verification, in-depth research and comparison. It is more efficient to compare different strategies in a standardized manner and then select a more effective combination for concentrated research.

Undoubtedly, ADSCs have a promising future, but their clinical transformation and application still face numerous obstacles, such as the source and collection site of donor cells, the applied dose and loading mode, and the composition and processing technology of biomaterials as scaffold substrates. To achieve the controlled sequential delivery of various BMs, complex designs are required, which may necessitate intricate, time-consuming and costly manufacturing processes. In addition, a successful clinical application must overcome a number of obstacles, including the establishment of regulatory guidelines, commercialization, mass production and cost control. The clinical transformation of BTE loaded with ADSCs remains underdeveloped. On the other hand, research on ADSCs promoting bone regeneration is predominantly macro-focused, and the underlying mechanism of ADSCs in bone regeneration remains unknown. The latter is especially important for the development and design of ADSC-loaded BTE, particularly the selection of scaffold materials and BMs, and must be explored further in the future.
